# Specific induction of right ventricular-like cardiomyocytes from human pluripotent stem cells

**DOI:** 10.1186/s13287-025-04656-0

**Published:** 2025-09-26

**Authors:** Yukihiro Saito, Kazufumi Nakamura, Yuki Katanosaka, Toshihiro Iida, Dai Kusumoto, Ryushi Sato, Riki Adachi, Satoshi Shimizu, Junko Kurokawa, Satoshi Akagi, Masashi Yoshida, Toru Miyoshi, Hiroshi Morita, Keiji Naruse, Mikako Nishida, Heiichiro Udono, Jianhua Zhang, Shinsuke Yuasa, Timothy J. Kamp, Hiroshi Ito

**Affiliations:** 1 Department of Cardiovascular Medicine, Okayama University Hospital, Okayama, Japan; 2 Department of Cardiovascular Medicine, Okayama University Faculty of Medicine, Dentistry and Pharmaceutical Sciences, Okayama, Japan; 3 Department of Cardiovascular Physiology, Okayama University Faculty of Medicine, Dentistry and Pharmaceutical Sciences, Okayama, Japan; 4 Department of Pharmacy, Kinjo Gakuin University, Nagoya, Japan; 5 Graduate School of Pharmaceutical Sciences, Kinjo Gakuin University, Nagoya, Japan; 6 Department of Cardiovascular Medicine, Okayama University Graduate School of Medicine, Dentistry and Pharmaceutical Sciences, Okayama, Japan; 7 Department of Biomedical Informatics and Molecular Biology, The Sakaguchi Laboratory, Keio University School of Medicine, Tokyo, Japan; 8 Department of Bio-Informational Pharmacology, School of Pharmaceutical Sciences, University of Shizuoka, Shizuoka, Japan; 9 Department of Chronic Kidney Disease and Cardiovascular Disease, Okayama University Faculty of Medicine, Dentistry and Pharmaceutical Sciences, Okayama, Japan; 10 Department of Cardiovascular Therapeutics, Okayama University Faculty of Medicine, Dentistry and Pharmaceutical Sciences, Okayama, Japan; 11 Department of Metabolic Immune Regulation, Okayama University Faculty of Medicine, Dentistry and Pharmaceutical Sciences, Okayama, Japan; 12 Department of Medicine, University of Wisconsin School of Medicine and Public Health, Madison, WI USA

**Keywords:** Human pluripotent stem cell-derived cardiomyocytes, Anterior second heart field, Right ventricle, Bone morphogenetic protein

## Abstract

**Background:**

Applications employing human pluripotent stem cell-derived cardiomyocytes (hPSC-CMs) require well-characterized, chamber-specific hPSC-CMs. Distinct first heart field (FHF) and second heart field (SHF) cardiac progenitor populations give rise to the left ventricular (LV) and right ventricular (RV) cardiomyocytes, respectively. This developmental difference in cardiomyocyte origin suggests that chamber-specific cardiomyocytes have unique characteristics. Therefore, efficient strategies to differentiate human pluripotent stem cells (hPSCs) specifically to LV-like or RV-like cardiomyocytes are needed and it is still unknown whether there is a phenotypic difference between LV-like cardiomyocytes and RV-like cardiomyocytes derived from hPSCs.

**Methods:**

An established hPSC cardiac differentiation protocol employing sequential GSK3β inhibition followed by Wnt inhibition (GiWi) was modified by addition of insulin or BMP antagonists during mesoderm formation. Cardiac progenitor populations were evaluated for FHF and SHF markers, and differentiated hPSC-CMs were characterized for chamber-specific markers.

**Results:**

The GiWi protocol produced mainly FHF-like progenitor cells that gave rise to LV-like cardiomyocytes. Inhibition of endogenous BMP signaling during mesoderm induction using insulin or BMP antagonists reduced expression of FHF markers and increased expression of SHF markers in cardiac progenitor cells. hPSC-CMs arising from the SHF-like progenitor cells showed an RV-like gene expression pattern and exhibited phenotypic differences in spontaneous contraction rate, Ca^2+^ transients, and cell size compared to control LV-like cardiomyocytes.

**Conclusion:**

This study establishes methodology to generate RV-like hPSC-CMs to support the development of disease modeling research using chamber-specific hPSC-CMs.

**Supplementary Information:**

The online version contains supplementary material available at 10.1186/s13287-025-04656-0.

## Introduction

The number of patients with heart disease continues to increase and cardiovascular disease remains the leading cause of death worldwide [1, 2]. Advances in the diagnosis and treatment of heart disease have identified a significant subset of patients with primarily right ventricular pathology, such as Brugada syndrome, arrhythmogenic right ventricular cardiomyopathy, certain forms of congenital heart disease, and right heart failure secondary to pulmonary arterial hypertension [3–7]. The pathophysiology is incompletely understood for these right ventricular diseases, and there are limited therapeutic options, in contrast to the range of therapies addressing diseases that primarily impact the left ventricle.

Several structural and functional features are known to differ between the left ventricle (LV) and the right ventricle (RV) [8]. During heart development, LV and RV arise from distinct populations of cardiac progenitor cells expressing distinct transcription factors [9]. Cardiac progenitor cells in the first heart field (FHF) are TBX5^+^/NKX2-5^+^ and form the linear heart tube which gives rise to primarily to the LV and atria. Cardiac progenitor cells in the anterior second heart field (SHF) are TBX5^−^/NKX2-5^+^, and the anterior SHF progenitors migrate into the outflow tract of the heart tube and grow primarily into RV. Cardiac progenitor cells in the posterior SHF are also TBX5^+^/NKX2-5^+^ and the progenitors migrate into the inflow tract of the heart tube and contribute to the atria [10, 11].

Disease modeling with human pluripotent stem cell-derived cardiomyocytes (hPSC-CMs) has been conducted for some right ventricular diseases [12–15]. However, it is unclear in those reports whether the hPSC-CMs were LV-like cardiomyocytes or RV-like cardiomyocytes. The chamber-specific and heart field-specific identity of hPSC-CMs is only beginning to be addressed by recent studies. Zhang et al.. generated *NKX2-5*^*TagRFP*^*/TBX5*^*Clover2*^ reporter human induced pluripotent stem cells (hiPSCs) to isolate NKX2-5^+^/TBX5^+^ FHF-like cells and NKX2-5^+^/TBX5^−^ SHF-like cells. When they examined cardiomyocytes derived from those progenitor cells, they concluded that cardiomyocytes from SHF-like cells were atrial-like cells [16]. Pezhouman et al.. also reported a method for inducing FHF-like cells and SHF-like cells separately using different CHIR 99,021 concentrations and seeding densities from HES3-*TBX5-TdTomato*^*+/W*^*/NKX2-5*^*eGFP/W*^ double reporter human embryonic stem cells (hESCs) [17]. They concluded that most of the induced cardiomyocytes from FHF-like and SHF-like cells were atrial-like cardiomyocytes. Andersen et al.. identified CXCR4 as a marker for SHF-like cells derived from pluripotent stem cells. They sorted CXCR4^+^ cardiac progenitor cells derived from hiPSCs; however, they did not indicate whether CXCR4^+^ cardiac progenitor cells give rise to ventricular myocytes or atrial myocytes [18]. In addition, Zhang et al.. induced SHF-like cells from hiPSCs using a fibroblast medium supplemented with basic fibroblast growth factor (bFGF), but the protocol was not for cardiomyocyte induction [19]. Recently, Yang et al. have separately induced FHF-like cells giving rise to LV-like cardiomyocytes and SHF-like cells giving rise to RV-like and atrial cardiomyocytes using different concentrations of BMP4 and Activin A [20]. However, the signaling pathways that separate the anterior SHF-like cells, which gives rise to RV-like cardiomyocytes, from the posterior SHF-like cells, which gives rise to atrial cardiomyocytes, are unknown, and cell sorting is required to selectively obtain RV-like cardiomyocytes. In addition, it is also unclear whether their method would be useful for hiPSCs.

In the present study, we develop methods to specifically differentiate human pluripotent stem cells (hPSCs) to RV-like and separately to LV-like cardiomyocytes and investigate the phenotypic differences between them.

## Materials and methods

### Human pluripotent stem cell culture

HiPSCs established from a healthy Japanese male donor (Supplemental Fig. S1A and B), 201B7 hiPSCs (RIKEN BRC, Tsukuba, Japan), and HES3-*TBX5-TdTomato*^*+/W*^*/NKX2-5*^*eGFP/W*^ reporter hESCs, a kind gift from Dr. Reza Ardehali, UCLA Geffen School of Medicine [17], were used in this study. HiPSCs were maintained on iMatrix-511 (Matrixome, Osaka, Japan)-coated 6-well plates (Corning, Glendale, Arizona, United States) in StemFit AK02N (Ajinomoto, Tokyo, Japan). HiPSCs were passaged every 7 days. HESCs were maintained on Matrigel Growth Factor Reduced Basement Membrane Matrix (Corning)-coated 6-well plates (Corning) in StemFlex Medium (Thermo Fisher Scientific, Waltham, MA, United States). HESCs were passaged every 3–4 days. StemPro Accutase Cell Dissociation Reagent (Thermo Fisher Scientific) was used for dissociation. 10 µmol/L Y27632 (Tocris, Bristol, United Kingdom) was used for improvement of cell survival for 24 h after seeding. Mycoplasma tests were performed for all the cell culture in this study.

### Generation of HiPSCs

An hiPSC line was generated from human peripheral blood mononuclear cells (PBMCs) collected from a 34-year-old healthy male according to the protocol published by Center for iPS Cell Research and Application, Kyoto University [21]. Peripheral mononuclear cells were purified with BD Vacutainer CPT (BD Biosciences, Franklin Lakes, NJ) according to the manufacturer’s instructions. First, 0.88 µg of pCXLE-hOCT3/4-shp53-F (#27077, Addgene, Watertown, MA, USA), 0.88 µg of pCXLE-hSK (#27078, Addgene), 0.88 µg pCXLE-hUL (#27080, Addgene) and 0.5 µg pCXWB-EBNA1 (#37624, Addgene) were electroporated into 3 × 10^6^ PBMCs with Nucleofector II (Lonza, Basel, Switzerland) and a Human T-cell Nucleofector Kit (Lonza) according to the manufacturer’s instructions. The program V‐024 was used. Then, 3 × 10^5^ cells were seeded onto six‐well plates covered with an SNL 76/7 feeder layer (#07032801, European Collection of Authenticated Cell Cultures, UK). The transfected cells were cultured in X‐vivo 10 Serum-free Hematopoietic Cell Medium (Lonza) supplemented with 10 ng/mL recombinant human Interleukin‐2 (PeproTech Inc., Rocky Hill, NJ) and 6.7 µl/well of Dynabeads Human T‐activator CD3/CD28 (Thermo Fisher Scientific). At 2 days, 4 days and 6 days after transfection, 1.5 mL of human ESC medium consisting of 80% DMEM/F12 (Thermo Fisher Scientific) supplemented with 20% knockout serum replacement (Thermo Fisher Scientific), 100 µmol/L 2-ME, MEM Non-Essential Amino Acids Solution (100X) (Thermo Fisher Scientific), and 10 ng/mL bFGF (Wako, Tsukuba, Japan) was added to each well without aspiration of the previous medium. The culture medium was then replaced with human ESC medium 8 days after transfection. The colonies were picked up 20 days after plating and expanded on iMatrix-511-coated plates with StemFit AK02N.

### Differentiation of hPSCs to cardiomyocytes

Cardiac differentiation was performed according to the protocol published by Lian et al. [22]. Cells were dissociated with StemPro Accutase Cell Dissociation Reagent at 37 °C for 5 minutes. HESCs were seeded on Matrigel-coated 24-well plates (Corning) in StemFlex Medium supplemented with 10 µmol/L Y-27,632. HiPSCs were seeded on iMatrix-511-coated 12-well plates (Corning) in StemFit AK02N supplemented with 10 µmol/L Y-27,632. The seeding densities were 1 × 10^5^ cells/cm^2^ for hESCs and 1.25–2.5 × 10^4^ cells/cm^2^ for hiPSCs. Cells were cultured for 4 days until differentiation started (day 0). On day 0, the medium was changed to RPMI 1640 Medium (Thermo Fisher Scientific) supplemented with B-27 Supplement minus insulin (Thermo Fisher Scientific) (RPMI/B27-insulin) or B-27 Supplement (Thermo Fisher Scientific) and 12 µmol/L CHIR 99,021 (SelleckChem, Houston, TX, United States), and the cells were treated in this medium for 24 h (day 1). On day 1, the medium was changed to RPMI/B27-insulin. To enhance cardiac differentiation, 50 µmol/L 2-O-α-D-glucopyranosyl-L-ascorbic acid (Tokyo Chemical Industry, Tokyo, Japan) was added on day 1 only for hiPSCs [23]. On day 3, the medium was changed to RPMI/B27-insulin supplemented with 5 µmol/L IWP2 (SelleckChem). On day 5, the medium was changed to 1 mL RPMI/B27-insulin. From day 7, the cells were cultured with RPMI/B27-insulin, and the cells were fed every other day. On day 12, the cells were washed with PBS (Thermo Fisher Scientific) and cultured with DMEM, no glucose supplemented with 8 mmol/L L-(+)-Lactic acid (Merck) [24]. The medium was changed every other day until day 17. After day 17, the cells were cultured with RPMI/B27 and fed every 2 to 3 days. For long-term culture, the cells were dissociated with TrypLE Select Enzyme (10X) (Thermo Fisher Scientific) at 37 °C for 15–20 min and seeded on Matrigel- or iMatrix-511-coated plates after day 35. Dorsomorphin 2HCl (SelleckChem) and DMH1 (SelleckChem) were used from day 0 to day 1.

### Knockdown of SMAD1

Forty nmol/L *SMAD1* or negative control siRNA (VHS41102, Thermo Fisher Scienrific) were transfected into undifferentiated cells using Lipofectamine RNAiMAX Transfection Reagent (Thermo Fisher Scientific) on cardiac differentiation day 0. The siRNA was removed on day 1. Gene expression patterns were evaluated on day 11.

### Quantitative polymerase chain reaction

Cells were lysed with TRIzol Reagent (Thermo Fisher Scientific). Total RNA was extracted using a PureLink RNA Mini Kit (Thermo Fisher Scientific). Complementary DNA was synthesized using SuperScript IV VILO Master Mix with ezDNase Enzyme (Thermo Fisher Scientific) as prescribed in the manual. PowerUp SYBR Green Master Mix (Thermo Fisher Scientific) and QuantStudio 1 (Thermo Fisher Scientific) were used for quantitative polymerase chain reaction (q-PCR). The q-PCR data were processed by the *ΔΔ*CT method. The q-PCR experiments were performed in technical duplicate. PCR primers were purchased from Integrated DNA Technologies (Coralville, IA, USA) and details are shown in Table 1. An RT^2^ Profiler PCR Array Human Fatty Acid Metabolism (Qiagen, Hilden, Germany) was used to examine fatty acid metabolism genes and the data were analyzed with the web-based tool of GeneGlobe Data Analysis Center (Qiagen). The mRNA expression levels of each fatty acid metabolism-related gene in the human heart were examined using the Human Protein Atlas (Version: 22.0) [25].

**Table 1:** PCR primers

Gene Symbol		Sequence	Product size (bp)	Annealing temperature (℃)
*CCK*	Forward	AAGCTCCTTCTGGACGAATGTC	96	60
	Reverse	AATCCATCCAGCCCATGTAGTC		
*CXCR4*	Forward	TCATCTCCAAGCTGTCACACTC	170	60
	Reverse	GTTCTCAAACTCACACCCTTGC		
*FGF10*	Forward	TTGAGAAGAACGGGAAGGTCAG	144	60
	Reverse	GTTTCCCCTTCTTGTTCATGGC		
*GAPDH*	Forward	CAACGACCACTTTGTCAAGCTC	144	60
	Reverse	TCTCTTCCTCTTGTGCTCTTGC		
*HAND1*	Forward	TCAAGGCTGAACTCAAGAAGGC	122	60
	Reverse	GGTGCGTCCTTTAATCCTCTTC		
*HCN4*	Forward	GGTGTCCATCAACAACATGG	66	60
	Reverse	GCCTTGAAGAGCGCGTAG		
*MYL2*	Forward	GTGCTGAAGGCTGATTACGTTC	121	60
	Reverse	TGTAGTCCAAGTTGCCAGTCAC		
*ISL1*	Forward	TCTGAGGGTTTCTCCGGATTTG	151	60
	Reverse	GCATTTGATCCCGTACAACCTG		
*NKX2-5*	Forward	GTCCCCTGGATTTTGCATTCAC	100	60
	Reverse	ATAATCGCCGCCACAAACTCTC		
*NPPA*	Forward	TCGATCTGCCCTCCTAAAAAGC	139	60
	Reverse	TCAGTACCGGAAGCTGTTACAG		
*NR2F2*	Forward	TCGCCTTTATGGACCACATACG	149	60
	Reverse	TTCCACATGGGCTACATCAGAG		
*SCN5A*	Forward	AGAAGATGGTCCCAGAGCAATG	131	60
	Reverse	AATCTGCTTCAGAACCCAGGTC		
*TBX1*	Forward	GTGGATGAAGCAAATCGTGTCC	197	60
	Reverse	TGAATCGTGTCTCCTCGAACAC		
*TBX5*	Forward	TCATCAGTACCACTCTGTGCAC	199	60
	Reverse	GAGTGCAGATGTGAACATTGGG		
*TNNT2*	Forward	TTCACCAAAGATCTGCTCCTCGCT	166	60
	Reverse	TTATTACTGGTGTGGAGTGGGTGTGG		
*SCN5A*	Forward	AGAAGATGGTCCCAGAGCAATG	131	60
	Reverse	AATCTGCTTCAGAACCCAGGTC		

### Bulk RNA-sequencing

Cells were lysed with TRIzol Reagent. Total RNA was extracted using a Monarch Total RNA Miniprep Kit (New England Biolabs, Ipswich, MA, United States). RNA sequencing was contracted to Rhelixa (Tokyo, Japan). FastQC was used for quality check, fastp for preprocessing, and Star-RSEM-DEseq2 pipeline for mapping, annotation, and extraction of expression variation genes. TPM was used for expression analysis. Correlation coefficients were calculated using Pearson correlation coefficient, and heatmaps were drawn using z score. RNA-seq data (GSE85728) from the left and right ventricles of postnatal day 0 mice (*n* = 3 in each) were reanalyzed and compared to data from iPS cell-derived cardiomyocytes.

### Immunocytochemistry

Cells were plated on iMatrix-511-coated plates and fixed in 4% paraformaldehyde (Nacalai, Kyoto, Japan) for 15 min. The cells were permeabilized with 0.1% Triton X-100 (Sigma-Aldrich, St. Louis, MO, United States)/PBS and blocked with 10% goat serum (Sigma-Aldrich). The primary and secondary antibodies were diluted in 0.1% Triton X-100/PBS with 5% goat serum. The cells were stained with Hoechst 33,342 (Thermo Fisher Scientific) at 1 µg/mL, mouse anti-cardiac troponin T (cTnT) monoclonal IgG1 (GTX28295, GeneTex, Irvine, CA, United States) diluted at 1:16000, mouse anti-α-actinin monoclonal IgG1 (A7811, Sigma-Aldrich) diluted at 1:1000, rabbit anti-MLC2v monoclonal IgG (ab92721, Abcam, Cambridge, United Kingdom) diluted at 1:500, rabbit anti-CXCR4 monoclonal IgG (ab181020, Abcam) diluted at 1:2000, rabbit anti-NKX2-5 monoclonal IgG (Cat #. 8972 S, Cell Signaling Technology, Danvers, MA, United States) diluted at 1:2500, mouse anti-TBX5 monoclonal IgG2a (Cat #. sc-515536, Santa Cruz Biotechnology, Dallas, TX, United States) diluted at 1:500, and rabbit anti-Na_v_1.5 polyclonal IgG (Cat #. ASC-013, Alomone labs, Jerusalem, Israel) diluted at 1:1000. The cells were incubated with a secondary antibody, goat anti-mouse or rabbit polyclonal IgG conjugated with iFluor 488 or 555 (AAT Bioquest, Sunnyvale, CA, United States) diluted at 1:1000, for 1 h at room temperature. The cells were then observed with IX71 (Olympus, Tokyo, Japan) or EVOS FL Auto (Thermo Fisher Scientific). The images were processed with ImageJ [36].

### Flow cytometry

Cells were dissociated with StemPro Accutase and stained with a LIVE/DEAD Fixable Dead Cell Stain Kit (Thermo Fisher Scientific). Then the cells were fixed with 1% paraformaldehyde for 15 min at room temperature. The cells were permeabilized with 90% cold methanol for 30 min on ice. The cells were then stained overnight at 4℃ with primary antibodies: mouse anti-cTnT monoclonal IgG1 (Cat #. GTX28295, GeneTex), 0.05 µg/100 µL; mouse anti-TBX5 monoclonal IgG2a (Cat #. sc-515536, Santa Cruz Biotechnology), 1:500 dilution; rabbit monoclonal Ig, 1:2500 dilution (Cat #. 8972 S, Cell Signaling Technology). Goat anti-mouse or rabbit polyclonal IgG conjugated with Alexa Fluor 488 or 647 diluted at 1:2000 was used as a secondary antibody. The cells were resuspended in 0.5% bovine serum albumin (Merck)/PBS. The cells were analyzed with an Attune NxT Flow Cytometer (Thermo Fisher Scientific).

### Western blot analysis

Using the cell lysis solution contained in the Minute Total Protein Extraction Kit for Animal Cultured Cells/Tissues (Invent Biotechnologies, Plymouth, MN, United States) with Protease Inhibitor Cocktail Set III (x100) (Fujifilm Wako Pure Chemical Corporation, Osaka, Japan) and Phosphatase Inhibitor Cocktail Solution II (×100) (Fujifilm Wako Pure Chemical Corporation), cells at day 3 of differentiation were lysed and proteins were extracted according to the manual. Protein concentration was measured with a Pierce BCA Protein Assay Kit (Thermo Fisher Scientific), iMark microplate reader and Microplate Manager 6 software (Bio-Rad, Hercules, CA, USA). Fifty micrograms of proteins combined with Laemmli sample buffer (Bio-Rad) were boiled at 95 °C for 5 min and then loaded onto 10% Mini-PROTEAN TGX Precast Gels (Bio-Rad). Proteins were transferred to nitrocellulose membranes. Blocking was performed using Tris buffered saline with Tween20 with 5% nonfat dry milk for 1 h at room temperature. The membranes were probed with the following antibodies overnight at 4 °C: anti-SMAD1 (Cat. #. 6944 S, Cell Signaling Technology) diluted at 1:1000, anti-phosphor-SMAD1/5 (Cat. #. 9516 S, Cell Signaling Technology) diluted at 1:1000, anti-SMAD2/3 (Cat. #. 8685 S, Cell Signaling Technology) diluted at 1:1000 and anti-phospho-SMAD2/3 (Cat. #. 8828 S, Cell Signaling Technology) diluted at 1:1000. A horseradish peroxidase-conjugated secondary antibody was used. To enhance the signal, Easy-Western II super (Beacle, Kyoto, Japan) was used according to the manual. Immunoblotted proteins were detected by ECL Prime Western blotting detection reagents and ImageQuant LAS4000 mini (GE Healthcare, Chicago, IL, USA).

### Measurement of cell area

Cardiomyocytes were immunolabeled with anti-cTnT antibody and the cell area was measured with Fiji [26].

### Measurement of intracellular Ca^2+^-transients

Cardiomyocytes were dissociated with TrypLE Select Enzyme (10X) (Thermo Fisher Scientific) and replated onto iMatrix-511-coated glass-based dishes (AGC Techno Glass, Shizuoka, Japan) at 1 × 10^5^/cm^2^ on day 20. On day 34, the cells were incubated with 20 µM Fura 2-AM (Dojindo Laboratories, Kumamoto, Japan) for 30 min in a CO_2_ incubator to load the indicator in the cytosol. The cells were washed twice with Tyrode’s solution. Fura 2-AM-loaded cells were alternately excited at 340 and 380 nm using a Lambda DG-4 Ultra High Speed Wavelength Switcher (Sutter Instruments) coupled to an inverted IX71 microscope with a UApo 20×/0.75 objective lens (Olympus). Fura 2 fluorescent signals were recorded (ORCA-Flash 2.8; Hamamatsu Photonics) during 0.2 Hz field stimulation and analyzed by a ratiometric fluorescence method using MetaFluor software (version 7.7.5.0; Molecular Devices).

### Evaluation of contraction speed of cardiomyocytes

Videos of the cardiomyocytes were taken using an OLYMPUS IX71 microscope and DPController (Olympus). The videos were analyzed with Musclemotion and Fiji according to the manual [26, 27].

### Electrophysiology

Action potentials from single human induced pluripotent stem cell-derived cardiomyocytes (hiPSC-CMs) were recorded at 36 ± 1 °C using the perforated patch-clamp technique [28]. This configuration was chosen to minimize the dialysis and washout of essential cytosolic components during recording. The cells were cultured as cell sheets on iMatrix‑511 silk-coated 35 mm dishes, and then dissociated into single cells a few days prior to patch‑clamp experiments. Recordings were performed on one of two available patch-clamp setups, each equipped with an Axopatch 200B amplifier (Molecular Devices, Sunnyvale, CA, Unitied States) and an inverted microscope (IMT-2 or IX-71, Olympus, Tokyo, Japan). The electrical signals were low-pass filtered at 2–5 kHz, digitized at a sampling rate of 1–2 kHz, and subsequently acquired and analyzed using pClamp10 software (Molecular Devices). The standard external solution contained the following (in mmol/L): 135 NaCl, 5.4 KCl, 1.8 CaCl₂, 0.53 MgCl₂, 0.33 NaH₂PO₄, 5.5 glucose, and 5 HEPES (pH 7.4 adjusted with NaOH). Borosilicate glass pipettes were filled with an internal solution containing (in mmol/L): 110 potassium aspartate, 30 KCl, 5 Mg-ATP, 5 creatine phosphate, 11 EGTA, 1 CaCl₂, and 5 HEPES. The pH was adjusted to 7.25 with KOH. To achieve the perforated patch configuration, amphotericin B (0.3 µg/ml) was freshly added to the internal solution. Recordings were commenced after the series resistance reached a stable value below 20 MΩ. For cardiomyocytes that did not exhibit spontaneous activity (i.e., quiescent cells), action potentials were elicited by injecting 2-ms depolarizing current pulses at 120% of the threshold intensity. To ensure data quality and reliability, only cells that produced stable and consistent action potentials for at least 10 consecutive traces were included in the final analysis [28].

### Evaluation of mitochondrial respiration

Oxygen consumption rate (OCR) were measured using the XF Cell Mito Stress Test on a Seahorse XFp Analyzer (Agilent Technologies, Santa Clara, CA, USA). Human iPSC-CMs were seeded in 96-well plates at 2 × 10^4 cells/well with RPMI/B27. Oligomycin (final concentration: 2.0 µmol/L), FCCP (final concentration: 0.5 µmol/L), and rotenone/antimycin A (final concentration: 0.5 µmol/L) were used for performing Cell Mito Stress Test.

### Statistical analysis

All data are expressed as means ± standard deviation (SD). Statistical analysis was performed by Student’s t-test using SPSS statistics 24 (IBM, Armonk, NY, United States). Values of *P* < 0.05 were considered to be significant.

### Ethics

All of the studies were approved by the Ethics Committee of Okayama University Graduate School of Medicine, Density, and Pharmaceutical Sciences, and written informed consent was obtained from the donor prior to the collection of PBMCs for the derivation of hiPSCs. The investigation also conformed to the principles outlined in the Declaration of Helsinki.

## Results

### Insulin promotes differentiation of SHF cardiac progenitors in the presence of biphasic Wnt signaling

**Fig. 1:**
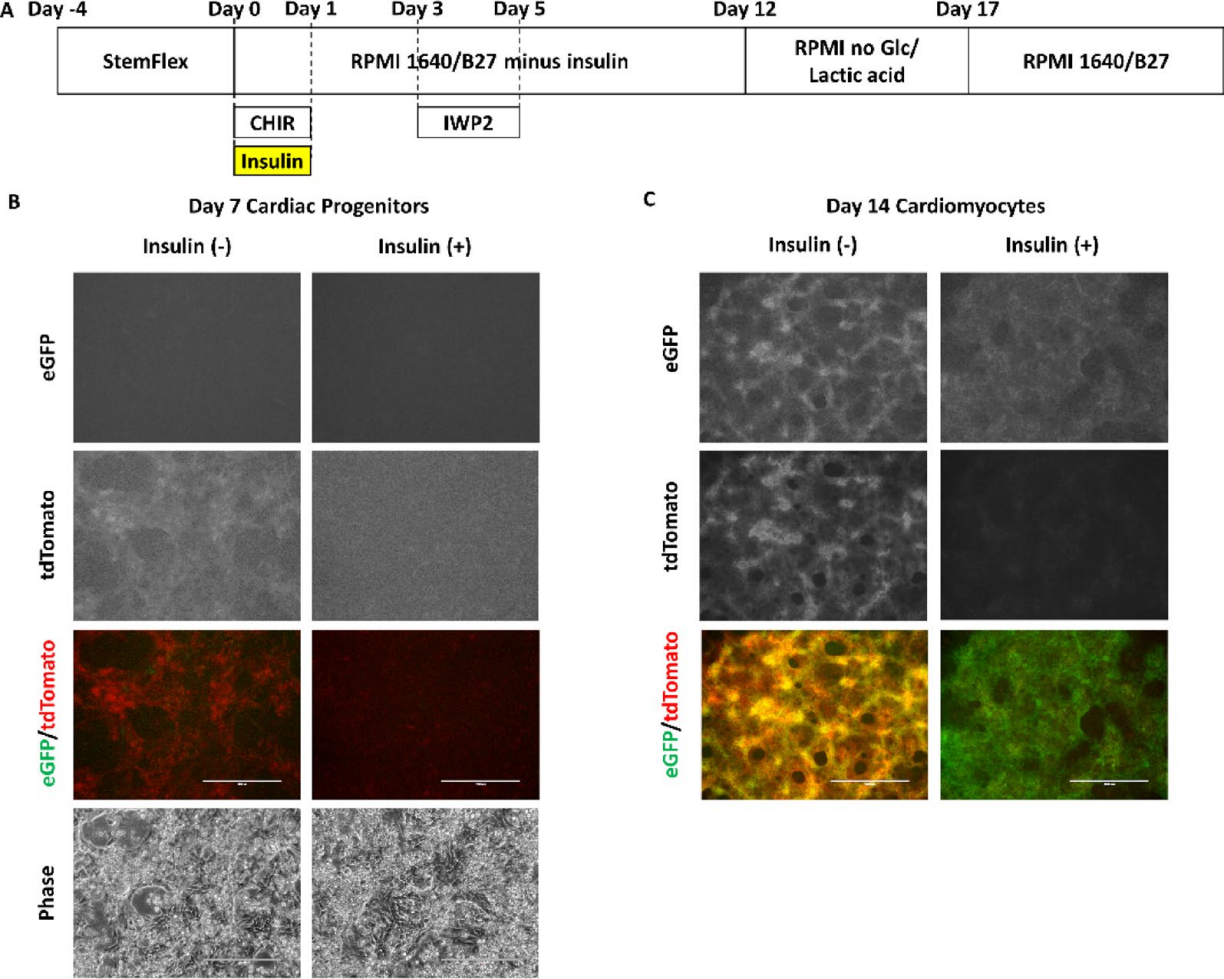
Insulin-containing medium on day 0 suppresses TBX5-tdTomato expression in HES3-*TBX5-TdTomato*^*+/W*^*/NKX2-5*^*eGFP/W*^ hESC-derived cardiac progenitor and cardiomyocytes. (A) Cardiac differentiation protocol in this section. B-27 supplement was used in place of B-27 supplement minus insulin on differentiation day 0. (B) NKX2-5-eGFP and TBX5-tdTomato expression on differentiation day 7 corresponding to the progenitor stage. The scale bars are 200 μm. (C) NKX2-5-eGFP and TBX5-tdTomato expression on differentiation day 14 corresponding to the myocyte stage. The scale bars are 1000 μm

Prior studies have demonstrated that insulin signaling inhibits the formation of cardiac mesoderm in some protocols using embryoid bodies, conditioned medium, and Activin A/BMP4 mediated differentiation [29, 30]; nevertheless, in a small molecule protocol using biphasic modulation of Wnt signaling (GiWi protocol), insulin did not inhibit cardiac differentiation [31]. However, prior studies did not exam the impact of insulin on the type of cardiac progenitors that form in the GiWi protocol. Using a HES3-*TBX5-TdTomato*^*+/W*^*/NKX2-5*^*eGFP/W*^ reporter hESC line that can identify FHF (TBX5-TdTomato^+^/NKX2-5-eGFP^+^) and SHF (TBX5-TdTomato^−^/NKX2-5-eGFP^+^) cardiac progenitors, we tested B-27 supplement with and without insulin on differentiation day 0 (Fig. 1A). TBX5-tdTomato was detected at the cardiac progenitor stage on day 7 by fluorescence microscopy in the absence of insulin but not in the presence (Fig. 1B). Subsequently, both control and insulin-treated cells expressed NKX2-5-eGFP, but the TBX5-tdTomato expression level in insulin-treated cells was lower than that in control cells on day 14, corresponding to the cardiomyocyte stage (Fig. 1C; Supplemental Videos 1 and 2). Since the concentration of insulin contained in the B-27 supplement is not disclosed, we tested several concentrations of insulin and found that 3 µg/mL insulin resulted in NKX2-5-eGFP expression without TBX5-tdTomato expression on day 13 consistent with the genesis of SHF-derived hPSC-CMs (Supplemental Fig. S2A).

**Fig. 2:**
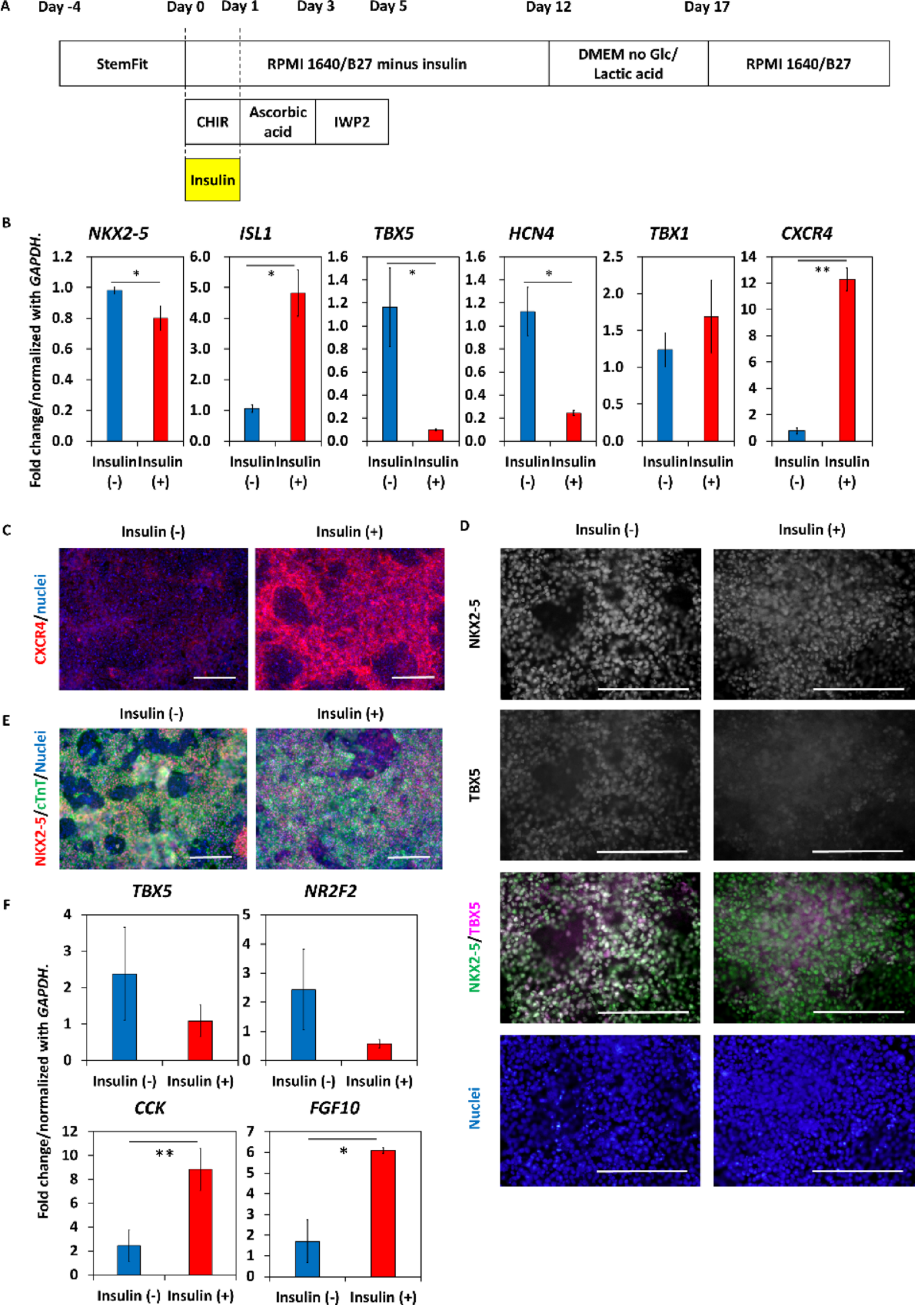
Insulin-containing medium on day 0 enhances SHF and RV marker expression in hiPSC-derived cells. (A) Cardiac differentiation protocol in this study. B-27 supplement was used in place of B-27 supplement minus insulin on differentiation day 0. (B) Gene expression profiles evaluated with quantitative PCR on day 7 (*n* = 3 in each). Data are shown as means ± standard deviation. *p < 0.05, **p < 0.01. (C) Immunostaining of CXCR4 on day 6. (D) Immunostaining of NKX2-5 and TBX5 on day 10. (E) Immunostaining of NKX2-5 and cardiac Troponin T (cTnT) on day 10. The scale bars are 200 μm. (F) Gene expression profiles of purified cardiomyocytes evaluated with quantitative PCR on day 20 (*n* = 3 in each). Data are shown as means ± standard deviation. *p < 0.05, **p < 0.01

We tested insulin treatment from day 0 to day 1 also in a reporterless hiPSC line to confirm the impact of insulin in an independent line (Fig. 2A). Insulin-treated cells showed lower mRNA expression of FHF markers (*TBX5* and *HCN4*) and higher mRNA expression of SHF markers (*ISL1* and *CXCR4*) than those in control cells on day 7 (Fig. 2B). Immunostaining for CXCR4 confirmed upregulation (Fig. 2C). Most of the control cells expressed TBX5, while TBX5 expression was greatly reduced in insulin-treated cells (Fig. 2D). Both control and insulin-treated cells expressed pan-cardiomyocyte markers, NKX2-5 and cTnT on day 10 (Fig. 2E). On day 20, purified cardiomyocytes have significantly higher mRNA levels of RV markers, *CCK* and *FGF10* [32, 33] (Fig. 2F). Furthermore, varying the timing of insulin treatment was tested, and not only day 0–1 treatment but also day 1–3 treatment induced cardiomyocytes with reduced TBX5 expression (Supplemental Fig. S5). Together, these findings suggest that the simple addition of insulin at the initiation of hPSC differentiation (day 0) in the GiWi protocol results in the formation of anterior SHF cardiac progenitors, the source of RV cardiomyocytes, in contrast to predominantly FHF cardiac progenitors in the absence of insulin as previously reported by Galdos et al. [34].

### Insulin suppresses phosphorylation of SMAD1/5

**Fig. 3:**
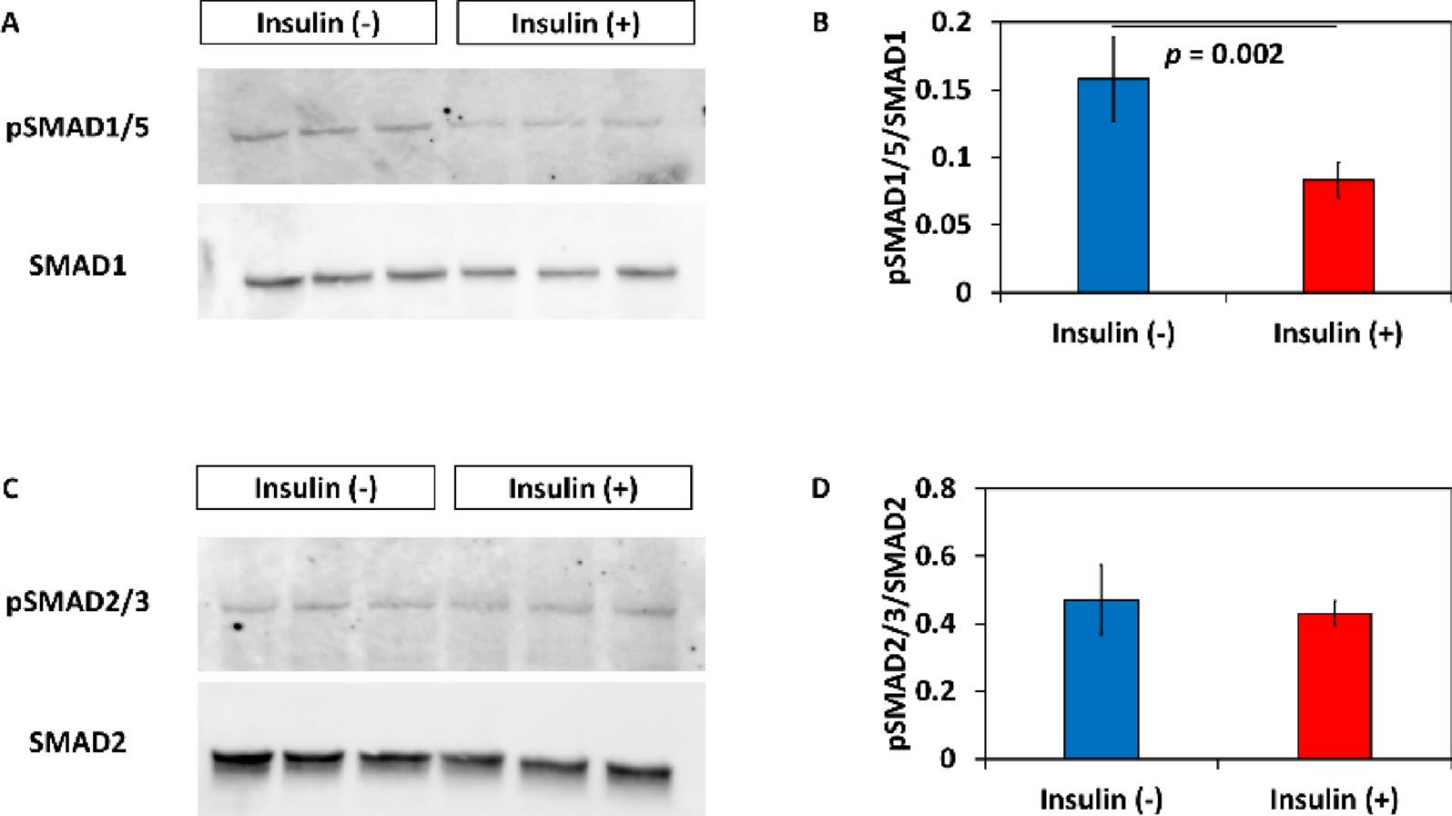
Effect of insulin on TGF-β/SMAD signaling during mesoderm induction. Phosphorylation of SMAD1/5 suggests activation of BMP signaling, and phosphorylation of SMAD2/3 suggests Activin signaling.　Phosphorylation of SMAD1/5 (A, B) and SMAD2/3 (C, D) was evaluated by western blotting (*n* = 3 in each). Data are shown as means ± standard deviation

Previous studies demonstrated that insulin inhibits cardiac differentiation of hPSCs in Activin A/BMP4-based protocols but not in the GiWi protocol [30, 31]. This suggests that insulin may modulate intrinsic Activin and/or BMP signaling in the GiWi protocol to regulate cardiac progenitor formation. Therefore, phosphorylation of SMADs was investigated which can be used as a readout for activation of Activin (SMAD2/3 phosphorylation) and BMP signaling (SMAD1/5 phosphorylation). Insulin added on differentiation day 0 suppressed phosphorylation of SMAD1/5 but not phosphorylation of SMAD2/3 measured on day 3 (Fig. 3A and B). This suggests that insulin inhibited activation of intrinsic BMP signaling during mesoderm induction.

### BMP receptor inhibitors induce SHF-like cells from reporterless HiPSCs

**Fig. 4:**
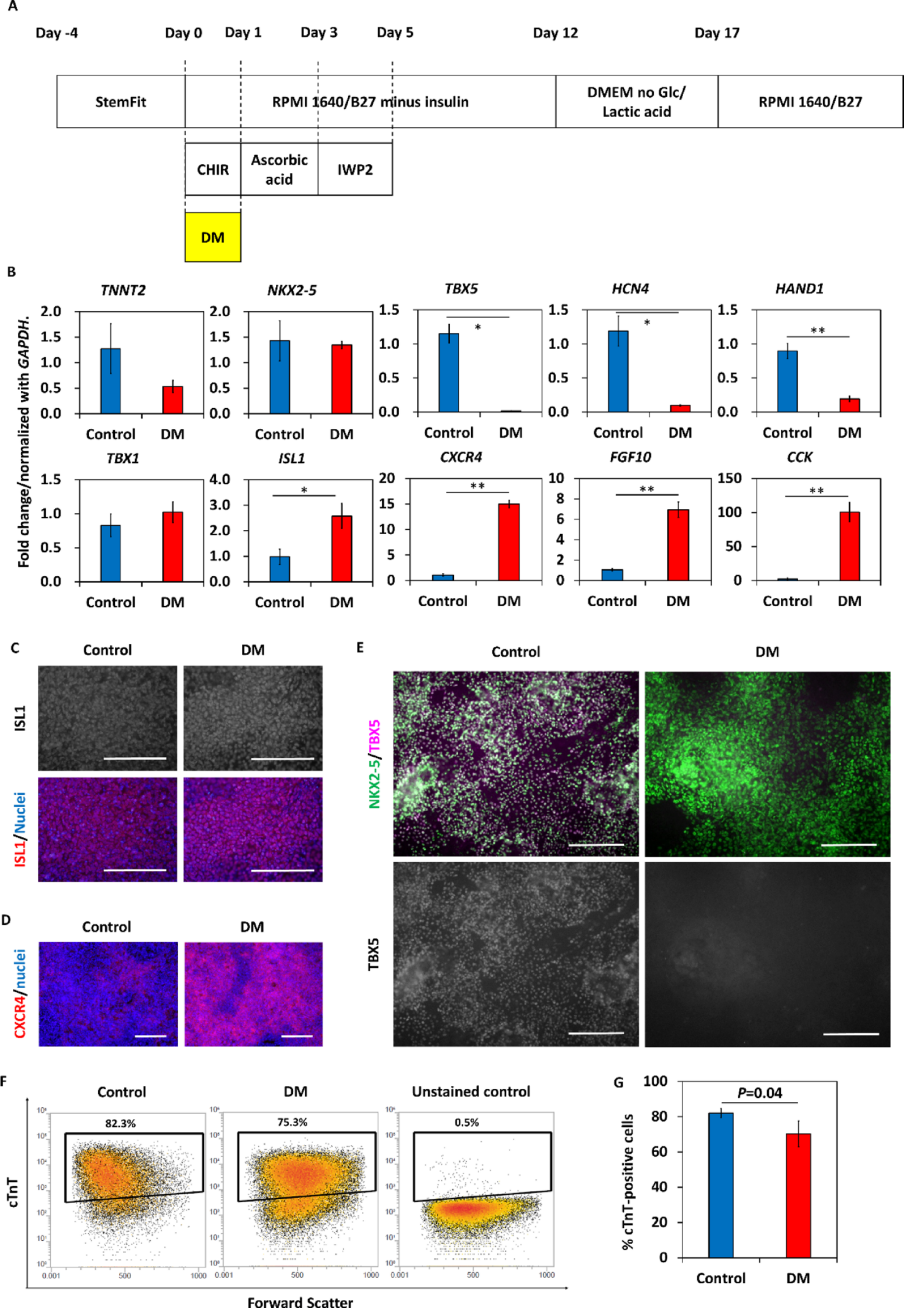
Treatment with dorsomorphin on day 0 induces SHF-like cells from hiPSCs. (A) Cardiac differentiation protocol in this section. Cells were treated with 1 µmol/L dorsomorphin on differentiation day 0. (B) Dorsomorphin downregulated the expression of first heart field (FHF) markers and upregulated the expression of second heart field (SHF) markers on day 7 (*n* = 3 in each). Data are shown as means ± standard deviation. *p < 0.01, **p < 0.001. (C) ISL was stained on day 6. (D) CXCR4 was stained on day 7. (E) Staining of NKX2-5 and TBX5 on day 10. (F) Flow cytometry data of cardiac troponin T (cTnT) on day 12. (G) Percentages of cTnT-positive cells (*n* = 4 in each). Data are shown as means ± standard deviation. The scale bars are 200 μm. DM: dorsomorphin

To determine if inhibiting BMP signaling directly could mimic the insulin effect on cardiac progenitor formation, we tested the effect of different concentrations of the BMP receptor inhibitor, dorsomorphin, added on differentiation day 0 (Fig. 4A). Dorsomorphin reduced the expression of FHF markers (*TBX5* and *HCN4*) and enhanced the expression of SHF markers (*TBX1*, *ISL1* and *CXCR4*) measured on day 7 in a dose-dependent manner (Fig. 4B, Supplemental Fig. S3A and B). However, 2 µmol/L dorsomorphin-treated cells did not show spontaneous contraction and did not survive metabolic selection using no glucose medium supplemented with lactic acid (Supplemental Fig. S3C). Therefore, we decided to use 1 µmol/L dorsomorphin for subsequent experiments. To evaluate the temporal pattern of changes in gene expression in response to dorsomorphin added on day 0, we performed quantitative PCR from day 0 to day 7 for a series of cardiomyocyte and cardiac progenitor genes (Supplemental Fig. S4). Dorsomorphin completely suppressed upregulation of *TBX5* until day 7. *ISL1* was highly expressed in control cells until day 5 then its expression level declined. On the other hand, gradual upregulation of *ISL1* was seen until day 7 in dorsomorphin-treated cells. The expression of *CORIN*, a FHF marker [16, 17], and the expression of *CD82*, a marker of cardiomyocyte-fated progenitors [35], were also suppressed in dorsomorphin-treated cells. Expression of *NKX2-5* and *TNNT2*, pan-cardiac markers, was delayed in dorsomorphin-treated cells.

Immunolabeling showed that both control and dorsomorphin-treated cells expressed ISL1 protein on day 6 and that CXCR4 protein was highly expressed in dorsomorphin-treated cells on day 7 (Fig. 4C and D). Both control and dorsomorphin-treated cells expressed NKX2-5 protein, while TBX5 protein expression was reduced in dorsomorphin-treated cells on day 10 (Fig. 4E). Flow cytometry data also showed that the proportion of NKX2-5-positive/TBX5-positive cells in the total NKX2-5-positive cells was lower in dorsomorphin-treated cells than in control cells: Control, 70.9 ± 5.0%; dorsomorphin-treated cells; 8.2 ± 1.7% (Supplemental Fig. S5A and B). On day 12, the percentages of cTnT-positive cells were 82.1 ± 2.6% in control cells and 70.4 ± 7.3% in dorsomorphin-treated cells (*p* = 0.04) (Fig. 4F and G). Interestingly, cTnT-positive cells in dorsomorphin-treated cells showed larger forward scatter than control cells did, suggesting that the size of cardiomyocytes in dorsomorphin-treated cells was larger than that in control cells (Fig. 4F). Different timing of dorsomorphin treatment was tested, and the addition of dorsomorphin on days 0, 1 or 3 of differentiation induced mainly TBX5-negative cardiomyocytes. (Supplemental Fig. S5C)

Dorsomorphin inhibits not only BMP signaling but also AMPK signaling. Therefore, DMH1, a selective inhibitor of the BMP receptor, was also tested. Treatment with DMH1 on differentiation day 0 also reduced TBX5 expression in a dose-dependent manner and induced NKX2-5-positive/cTnT-positive cardiomyocytes (Supplemental Fig. S6A and B). Treatment with 0.5 µmol/L DMH1 upregulated CXCR4 expression on day 7 (Supplemental Fig. S6C).

### RV-like cardiomyocytes arise from SHF-like cells

**Fig. 5:**
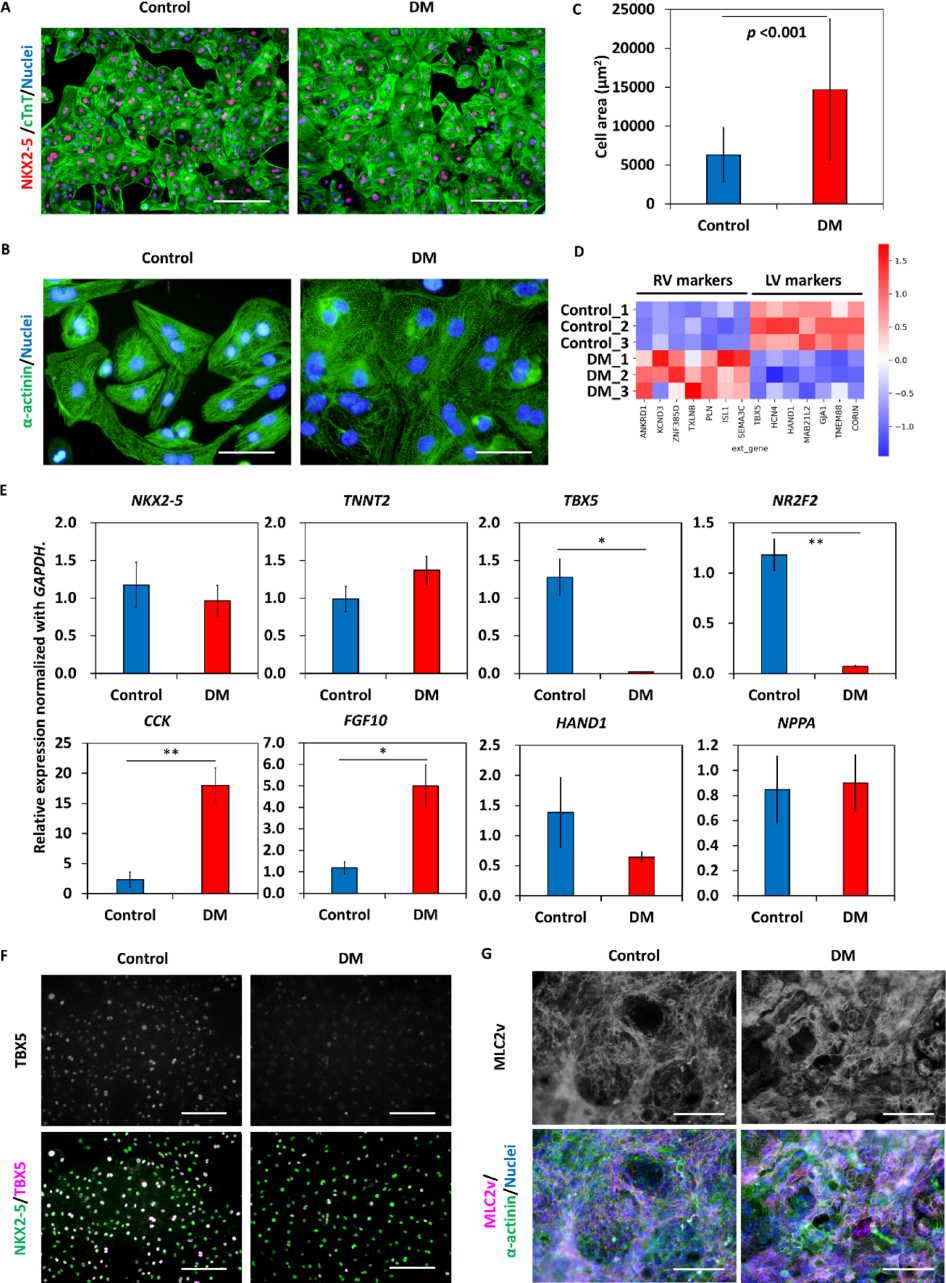
Addition of dorsomorphin on day 0 induces RV-like cardiomyocytes from hiPSCs. (A) Immunostaining of cardiac Troponin T (cTnT) and NKX2-5 in day 20 cardiomyocytes. The scale bars are 100 μm. (B) Immunostaining of α-actinin in day 50 cardiomyocytes. The scale bars are 25 μm. (C) Comparison of cardiomyocyte area (*n* = 153 and 146, respectively). Data are shown as means ± standard deviation. (D) Heatmap of differentially expressed left ventricular and right ventricular marker genes in cardiomyocytes. (E) Gene expression profiles evaluated with qPCR on day 20 (*n* = 3 in each). Data are shown as means ± standard deviation. *p < 0.05, **p < 0.01. (F) Immunostaining of NKX2-5/TBX5 in differentiation day 20 cardiomyocytes. (G) Immunostaining of MLC2v and α-actinin on differentiation day 35. The scale bars are 100 μm. DM: dorsomorphin

Cardiomyocytes were purified by culture in glucose-free/lactic acid supplemented medium [24]. Most of the cells in both the control and dorsomorphin-treated groups expressed NKX2-5 and cTnT as shown in Fig. 5A. Cardiomyocytes arising from dorsomorphin-treated cells appeared larger than control cardiomyocytes (Fig. 5B and C). The induction of different subtypes of cardiomyocytes was comprehensively investigated by bulk RNA-seq analysis (Supplementary Fig. S7A and B). The RNA-seq data showed that control cardiomyocytes highly expressed LV markers and cardiomyocytes derived from dorsomorphin-treated cells highly expressed RV markers (Fig. 5D). Public bulk RNA-seq data comparing the LV and RV of P0 mice was reanalyzed and compared it with our data from hiPSC-CMs. Several genes that were significantly upregulated in the mouse RV were also significantly upregulated in dorsomorphin-treated cell-derived cardiomyocytes, and conversely, genes that were downregulated in the mouse RV were also downregulated in dorsomorphin-treated cell-derived cardiomyocytes (Supplementary Fig. S7C and D). Quantitative PCR data confirmed that there was no significant difference between the two groups in expression of cardiomyocyte markers, *NKX2-5* and *TNNT2* and a working cardiomyocyte marker, *NPPA* [36]. However, the expression levels of *CCK* and *FGF10* (RV markers) were significantly upregulated and the expression levels of *TBX5* (LV and atrial marker) and *NR2F2* (atrial and nodal marker) were significantly downregulated in hPSC-CMs derived from dorsomorphin-treated cells. The expression level of *HAND1* (LV marker) trended to be lower with dorsomorphin treatment (Fig. 5E). Immunolabeling also showed less TBX5 expression in cardiomyocytes from dorsomorphin-treated cells (Fig. 5F). At a later differentiation stage (day 35), both groups of cardiomyocytes expressed MLC2v protein, a ventricular-type myosin light chain isoform (Fig. 5G).

Additionally, cardiomyocytes derived from DMH1-treated cells also expressed MLC2v, a ventricular marker protein (Supplemental Fig. S6D). Cardiomyocytes derived from DMH1-treated cells showed lower mRNA level of *TBX5* and higher mRNA level of *FGF10* (Supplementary Fig. S6E). As shown in Supplemental Fig. S8A-D, similar results were obtained using commercially available hiPSC line, 201B7.

To validate the results from BMP signaling inhibitors, genetically inhibition of BMP signaling was performed. *SMAD1* siRNA or negative control siRNA was transfected at differentiation day 0. A significant decrease in *SMAD1* mRNA level was observed 2 days after transfection of *SMAD1* siRNA: negative control siRNA, 1.012; *SMAD1* siRNA, 0.723 relative fold (*p* = 0.009, *n* = 3 in each). Reduced TBX5 expression level and increased *FGF10* and *CCK* transcription levels were observed in cardiomyocytes derived from cells treated with *SMAD1* siRNA compared to negative control siRNA-treated cell-derived cardiomyocytes at differentiation day 11 (Fig. S9A, B).

### Phenotypic characteristics of RV-like cardiomyocytes derived from HiPSCs

**Fig. 6:**
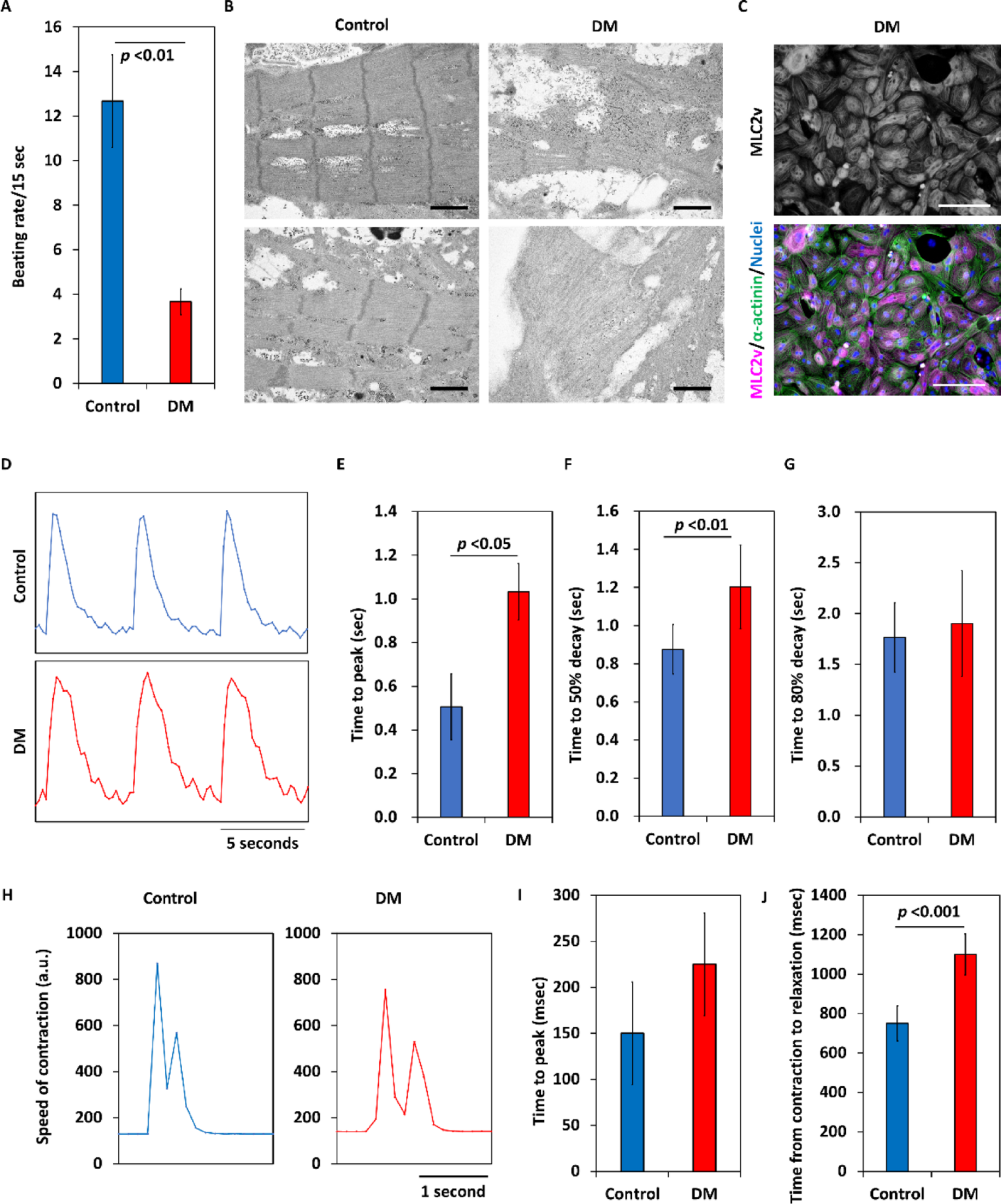
Phenotypic characteristics of RV-like cardiomyocytes derived from hiPSCs. (A) Spontaneous contraction frequency of day 46 cardiomyocytes (*n* = 3 in each). Data are shown as means ± standard deviation. (B) Transmission electron microscopic images of cardiomyocytes on differentiation day 50. Scale bars are 1 μm. (C) Immunostaining of MLC2v and α-actinin on day 52. The scale bars are 100 μm. (D) Representative calcium transients recorded from control (upper) and dorsomorphin-treated cell-derived cardiomyocytes (bottom) on day 34. (E) Time to peak calcium transient, (F) time to 50% decay and (G) time to 80% decay (*n* = 7 in each). Data are shown as means ± standard deviation. (H) Speed of contraction of cardiomyocytes. (I) Time to peak contraction (*n* = 5 in each). Data are shown as means ± standard deviation. (J) Time from start of contraction to end of relaxation (*n* = 5 in each). Data are shown as means ± standard deviation. DM: dorsomorphin

**Fig. 7:**
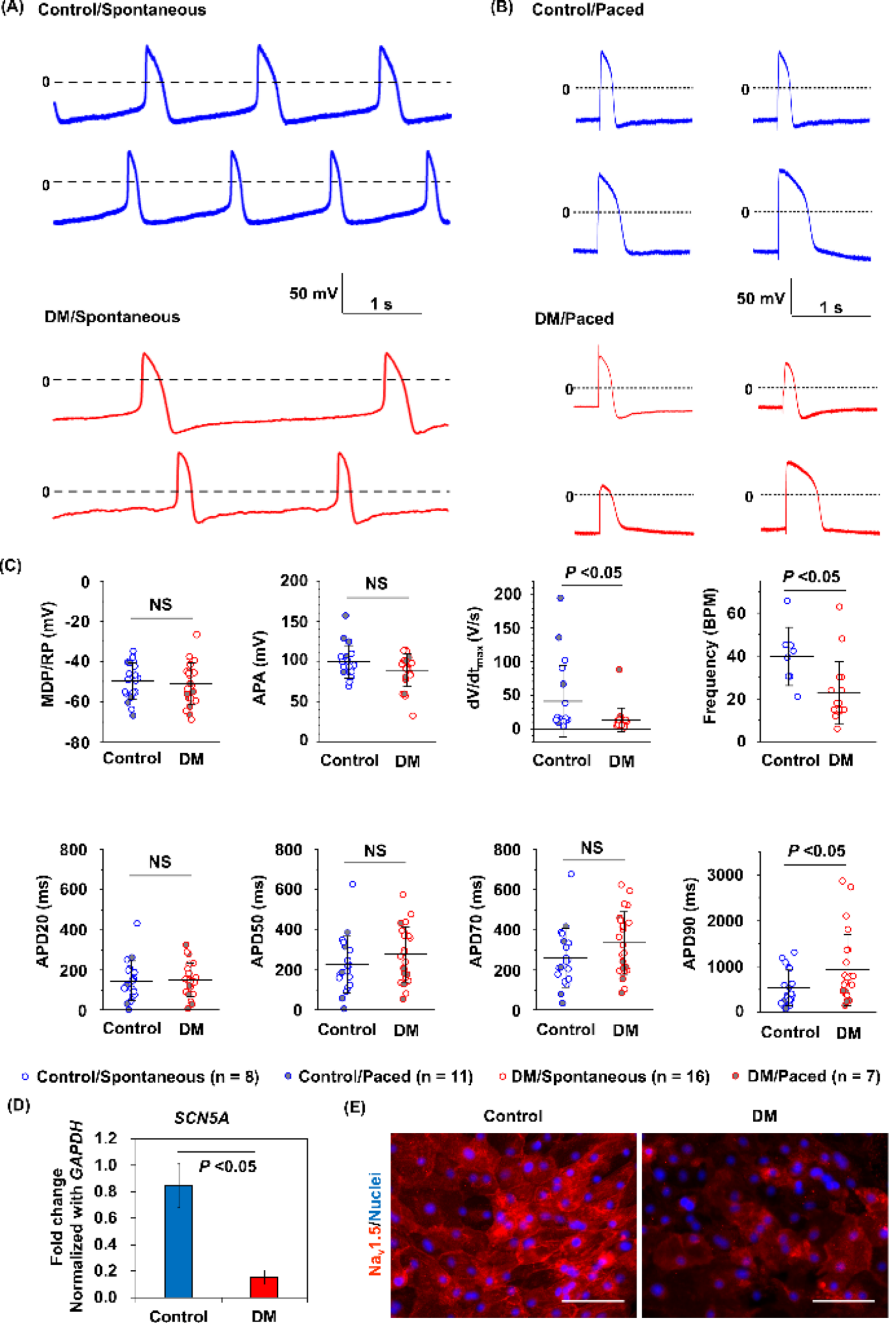
Differences in action potential configurations using the perforated patch-clamp technique. (A) Representative action potential configurations in spontaneous beating cardiomyocytes. (B) Representative action potential configurations in paced cardiomyocytes. (C) Comparison of action potential parameters. Data are shown as means ± standard deviation. (D) Comparison of *SCN5A* mRNA expression (*n* = 3 in each). Data are shown as means ± standard deviation. (E) Immunostaining of Na_v_1.5. The scale bar is 100 μm. DM: dorsomorphin. MDP/RP: maximum diastolic potential/resting potential. APA: action potential amplitude. APD20: action potential duration at 20% repolarization. APD50: action potential duration at 50% repolarization. APD70: action potential duration at 70% repolarization. APD90: action potential duration at 90% repolarization

Spontaneous contracting rates of cardiomyocytes from dorsomorphin or DMH1-treated cells were lower than those of control cardiomyocytes (Fig. 6A; Supplemental Videos 3 and 4; Supplemental Fig. S6F; Fig. 7C). Electron microscopic findings showed that dorsomorphin-treated cell-derived cardiomyocytes had poor sarcomere maturation, even though the mature ventricular marker MLC2v was already expressed well (Fig. 6B and C). Calcium transients in both groups of cardiomyocytes are shown in Fig. 6D. Time to peak and time to 50% decay of the calcium transient were greater in cardiomyocytes from dorsomorphin-treated cells than in control cardiomyocytes (Fig. 6E-G). The contraction speeds of cardiomyocytes are shown in Fig. 6H. Time to peak contraction tended to be longer in cardiomyocytes from dorsomorphin-treated cells than in control cardiomyocytes (Fig. 6I). The time from the start of contraction to relaxation was significantly longer in cardiomyocytes from dorsomorphin-treated cells than in control cardiomyocytes (Fig. 6J). In addition, dorsomorphin-treated cell-derived cardiomyocytes showed lower dV/dt_max_ and longer action potential duration at 90% repolarization than control cardiomyocytes (Fig. 7A-C). The expression level of sodium channels associated with the upstroke velocity of the action potential was significantly lower in dorsomorphin-treated cell-derived cardiomyocytes than in control cardiomyocytes (Fig. 7D and E).

### Differential gene expression related to lipid metabolism

**Fig. 8:**
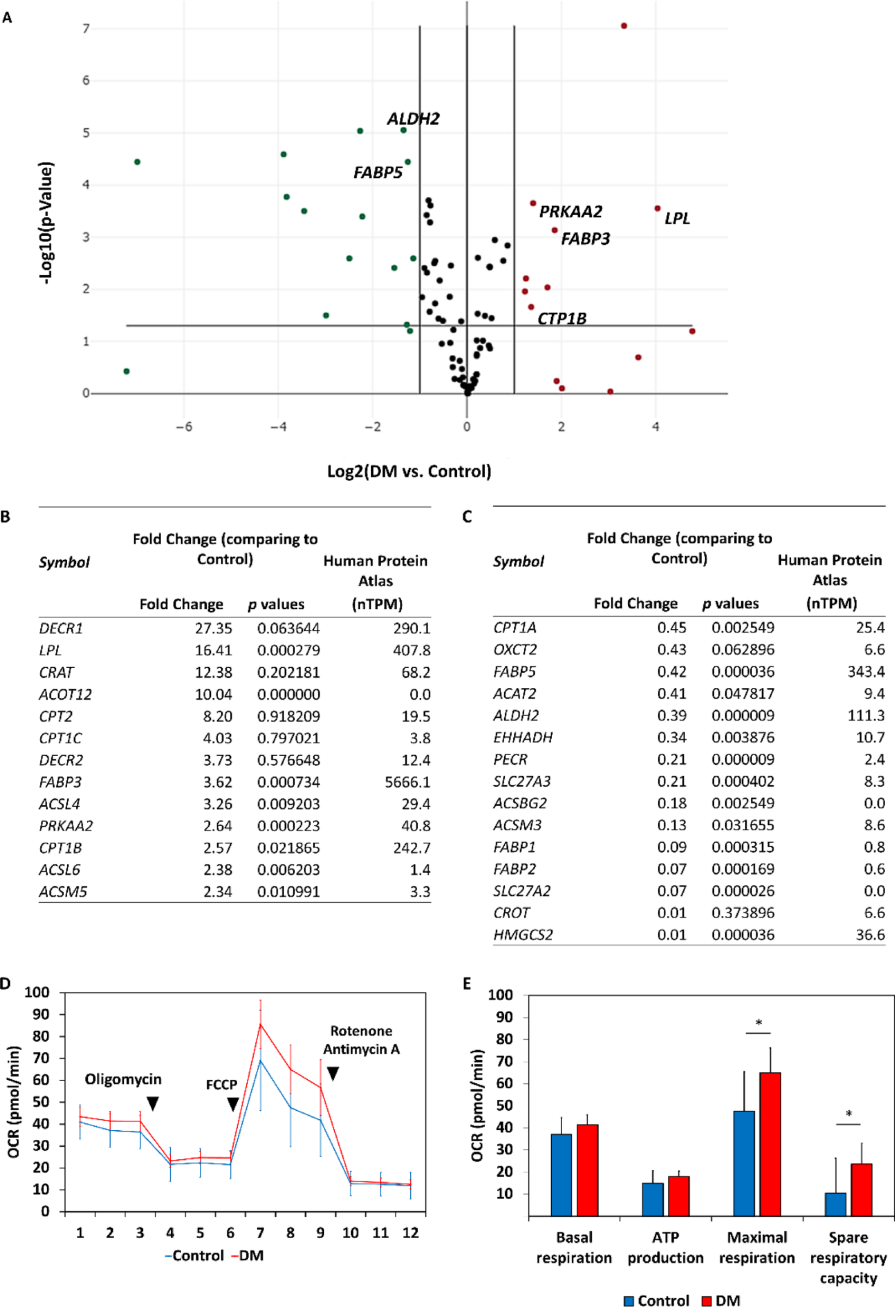
Differences in metabolism in hiPSC-CMs on day 20. (A) Scatterplots showing expression profiles of 84 genes related to fatty acid metabolism. Red and green dots denote significantly upregulated and downregulated genes (*n* = 3 in each group, *p* < 0.05 and fold change > 2 or < 0.5), respectively. (B) List of genes with more than 2-fold higher expression than control cardiomyocytes. (C) List of genes whose expression is less than half that of control cardiomyocytes. The rightmost column shows RNA expression levels (nTPM) in the heart as listed in the Human Protein Atlas (version 21.1, https://www.proteinatlas.org/). (D) Oxygen consumption rate (OCR) of hiPSC-CMs. (E) OCR parameters representing mitochondrial respiration. (*n* = 43 in control group and *n* = 45 in DM group, *p < 0.01). DM: dorsomorphin

Since differential expression of genes related to fatty acid metabolism between RV myocytes and other chamber myocytes has recently been reported [37], we compared the expression profiles of genes related to fatty acid metabolism with qPCR array. The expression levels of *LPL* [38], *FABP3* [39], *PRKAA2* [40] and *CPT1B* [41] genes involved in cardiac fatty acid metabolism were higher in dorsomorphin-treated cell-derived cardiomyocytes than those in control cardiomyocytes (Fig. 8A-C). OCR, indicative of mitochondrial respiration as measured by the Flux Analyzer, showed that dorsomorphin-treated cell-derived cardiomyocytes had higher maximal respiration and spare respiratory capacity than control cardiomyocytes did (Fig. 8D and E).

## Discussion

In this study, we succeeded in robust induction of anterior SHF-like cells giving rise to RV-like cardiomyocytes from hPSCs by modifying the GiWi protocol.

Using a HES3-*TBX5-TdTomato*^*+/W*^*/NKX2-5*^*eGFP/W*^ reporter hESC line, we found that adding insulin on day 0 of differentiation in the GiWi protocol with optimized cardiac differentiation efficiency yields cardiac progenitor cells and cardiomyocytes with low TBX5 expression. Since insulin was predicted to modulate BMP or Activin signaling based on the report of Lian et al. [31], we examined SMAD phosphorylation and found that SMAD1/5 phosphorylation, which suggests BMP signaling activity, was attenuated. The mechanism by which insulin inhibits SMAD1/5 phosphorylation is unknown, but it has been reported that IGF and FGF antagonize BMP signaling [42–44].

Simply adding insulin, dorsomorphin or DMH1 on differentiation day 0 in the GiWi protocol suppressed FHF-like cell induction and promoted SHF-like cell induction. Pezhouman et al.. showed that different combinations of seeding density and Gsk3 inhibitor CHIR 99,021 concentration on day 0 resulted in TBX5^+^/NKX2-5^+^ FHF-like or TBX5^−^/NKX2-5^+^ SHF-like cell induction from the HES3-*TBX5-TdTomato*^*+/W*^*/NKX2-5*^*eGFP/W*^ reporter hESC line [17]. Therefore, the fate of FHF or SHF can be determined at the mesoderm induction stage in hPSC cardiac differentiation. This is consistent with the phenomenon observed in a mouse embryo [9].

Andersen et al.. demonstrated that treatment with a BMP signaling inhibitor (dorsomorphin or DMH1) during the gastrulation stage decreased FHF-like cell induction but not SHF-like cell induction from *Hcn4-GFP*; *Tbx1-Cre*,* RFP* mouse embryonic stem cells [18]. Recently, Yang et al.. have reported that lower concentration of exogenous BMP4 and Activin A treatment induced both of anterior and posterior SHF-like populations that gave rise to RV-like and atrial-like cardiomyocytes, respectively [20]. Our study demonstrated that inhibition of BMP signaling during mesoderm induction using GiWi protocol suppressed TBX5 upregulation and induced anterior SHF-like cells rather than posterior SHF-like cells.

SHF-like cells induced in our study gave rise to cardiomyocytes showing downregulation of LV, atrial and nodal myocyte markers and upregulation of an RV myocyte marker. Induction of SHF-like cells has been reported so far, but it has been concluded that atrial myocytes were mainly generated from the induced SHF-like cells [16, 17]. Therefore, there is novelty in the induction of RV myocytes in our study. In our study, a glucose-free/lactic acid-supplemented medium was used to remove non-cardiomyocytes [24], and that might have promoted cardiomyocyte maturation resulting in expression of the ventricular myocyte marker MLC2v [45, 46]. Additionally, the purity of cTnT-positive cardiomyocytes from SHF-like cells was relatively high at approximately 70% compared to that in previous studies [16, 18]. Since SHF-like progenitor cells and RV-like cardiomyocytes were efficiently obtained only by exchanging the medium in this study, our method is simpler than the method requiring cell sorting using antibodies for cell surface markers or reporter genes [16, 17]. The metabolic selection is widely used for purification of hPSC-CMs and was useful for purification of both LV-like and RV-like hPSC-CMs. However, David et al.. recently reported that this method is useful as an ischemia model, and caution may be needed in cardiomyocyte purification by this method [47].

Interestingly, RV-like cardiomyocytes derived from hiPSCs exhibited several distinct phenotypic characteristics compared to control LV-like cardiomyocytes: slower spontaneous contraction rate, larger cell size, poorer sarcomere maturation, and sluggish calcium transients and contraction/relaxation speed. Peshoumen et al.. also reported that NKX2-5-positive/TBX5-negative SHF-derived cardiomyocytes from the hESC *TBX5-TdTomato*^*+/W*^*/NKX2-5*^*eGFP/W*^ reporter line showed poorer sarcomere organization and calcium handling [48].

The low spontaneous contraction frequency of RV-like cardiomyocytes might be due to low HCN4 expression level compared to that in control LV-like cardiomyocytes. HCN4 is a FHF marker in the early stages of cardiac development, but as development progresses, its expression is localized in the conduction system including the sinus node [11, 49]. Also in hPSC-CMs, HCN4 expression gradually decreases along with maturation [50]. *HCN4* encodes an HCN channel that generates a pacemaker current, inducing diastolic depolarization [51]. This may explain the low frequency of spontaneous contraction of RV-like cardiomyocytes.

It is unclear why cardiomyocytes were larger and sarcomere maturation was poorer in RV-like cardiomyocytes than in LV-like cardiomyocytes even though both cell types expressed one of the mature ventricular marker MLC2v. Kathiriya et al.. generated hiPSCs with *TBX5* heterozygous or homozygous loss of function mutation and reported that the lower the expression level of TBX5 was, the larger was the hiPSC-CM size and the less was myofilament organization. In other words, these phenomena are thought to be caused by a low expression level of TBX5. Additionally, it was reported that RV cardiomyocytes are larger in cell size than LV cardiomyocytes in the immature heart [52]. In vivo, fetal-specific hemodynamics may influence the increase in RV cardiomyocyte size. However, it is an interesting finding that there is a difference in hPSC-CM size in vitro, where hemodynamic involvement is absent.

Differences between LV and RV cardiomyocytes regarding Ca^2+^ transients and contraction/relaxation have been reported, but there is no unified view and they may vary depending on the developmental stage and species [8, 53]. In this study, RV-like cardiomyocytes were characterized by a slower nature of these phenotypes. Whether this is a difference between LV and RV or a difference in maturity needs to be further investigated.

Pathological studies using hiPSC-CMs have been conducted for various forms of heart disease. Results using patient-specific hiPSC-CMs have been reported for Brugada syndrome [54] and arrhythmogenic right ventricular cardiomyopathy [12], which predominantly impact the RV. However, it was not stated in those reports whether the hiPSC-CMs utilized corresponded to LV-like cardiomyocytes or RV-like cardiomyocytes. Since the subtypes of hiPSC-CMs can vary between cell lines and differentiation protocols, ambiguity about cardiomyocyte subtypes can have a significant impact on reproducibility in disease modeling studies and drug response tests [22, 55, 56]. In our study, RV-like cardiomyocytes from hiPSCs showed some different phenotypic characteristics. Disease-specific iPS cell-derived RV-like cardiomyocytes are expected to be more likely than LV-like cardiomyocytes to show characteristics of diseases in which the RV primarily manifests the pathology.

The low maturity of the sarcomere structure of RV-like cardiomyocytes may make it difficult to characterize the disease [57, 58]. Recently, it has been reported that RV cardiomyocytes have higher expression levels of genes related to fatty acid metabolism than other chamber cardiomyocytes do [37]. Also in our study, the expression levels of some genes involved in cardiac fatty acid metabolism were higher in RV-like cardiomyocytes than those in LV-like cardiomyocytes. In other words, it may be possible to target fatty acid metabolism in order to mature RV-like cardiomyocytes derived from hPSCs. A shift from glucose metabolism to fatty acid metabolism has been reported as a method for promoting the maturation of hPSC-CMs [46, 57, 58]. The method may also be useful for promoting maturation and myofilament organization of RV-like cardiomyocytes.

There are some limitations in this study. Progenitor cells were not sorted and their fates were not strictly tracked in this study. Using a HES3-*TBX5-TdTomato*^*+/W*^*/NKX2-5*^*eGFP/W*^ reporter hESC line, TBX5-tdTomato signal was not detectable with a fluorescent microscopy from differentiation day 0 to day 14. It is already known that TBX5-negative/NKX2-5-positive RV cardiomyocytes arise from TBX5-negative/NKX2-5-positive anterior SHF progenitor cells in vitro and in vivo, and our results do not contradict them [20, 59]. Furthermore, our study did not examine whether RV-like cardiomyocytes are a uniform cell population or whether RV working cardiomyocytes and outflow tract cells are intermixed as Schmidt et al.. suggested [60]. Schmidt et al. reported that delayed addition of retinoic acid can separate RV fate from the outflow tract fate and atrial fate. RV-like cardiomyocytes generated using our protocol expressed *NPPA* and *MYL2* at levels comparable to those of LV-like cardiomyocytes, suggesting that RV-like cardiomyocytes are mainly working cardiomyocytes, not outflow tract cells. Additionally, since this study did not compare the characteristics of LV and RV cardiomyocytes isolated from fetal human hearts with LV-like and RV-like hiPSC-CMs, further investigation is needed to determine whether hiPSC-CMs are strictly left or right ventricular.

## Conclusions

We have established a method for inducing anterior SHF-like progenitor cells giving rise to RV-like cardiomyocytes from hPSCs by inhibition of BMP signaling at the mesoderm induction stage in the GiWi protocol, which is a commonly used protocol worldwide. Since LV-like cardiomyocytes and RV-like cardiomyocytes can be separately induced by this method and they exhibit different characteristics, this method is expected to be applied to pathological models of diseases primarily impacting the RV.

## Supplementary Information

Below is the link to the electronic supplementary material.

Supplementary Material 1: Video 1. Cardiomyocytes derived from HES3-TBX5-TdTomato+/W/NKX2-5eGFP/W human embryonic stem cells on day 16. B-27 supplement minus insulin was used on differentiation day 0.

Supplementary Material 2: Video 2. Cardiomyocytes derived from HES3-TBX5-TdTomato+/W/NKX2-5eGFP/W human embryonic stem cells on day 16. B-27 supplement containing insulin was used on differentiation day 0.

Supplementary Material 3: Video 3. Control cardiomyocytes derived from human induced pluripotent stem cells.

Supplementary Material 4: Video 4. Cardiomyocytes derived from human induced pluripotent stem cells. Dorsomorphin was added on differentiation day 0.

Supplementary Material 5: Figure S1. Characterization of human pluripotent stem cells generated in our laboratory. (A) Immunostaining of undifferentiated cell markers expressed in hiPSCs. Scale bars are 100 µm. (B) Hematoxylin eosin staining of teratoma tissue derived from hiPSCs. The scale bar is 400 µm. Figure S2. (A) Treatment with 3 µg/mL insulin on differentiation day 0 results in low TBX5-tdTomato expression in cardiomyocytes on day 13. The scale bar is 400 µm. Figure S3. Treatment with different concentrations of dorsomorphin on differentiation day 0 induces second heart field-like cells from human induced pluripotent stem cells. (A) Dorsomorphin downregulated the expression of first heart field (FHF) markers and upregulated the expression level of second heart field (SHF) markers on day 7 (n = 1 in each). (B) Dorsomorphin-treated cells expressed NKX2-5, but TBX5 expression was suppressed on differentiation day 10. The scale bar is 100 µm. (C) Phase contrast images of purified cardiomyocytes on differentiation day 20. DM: dorsomorphin. Figure S4. Comparison of gene expression profiles between control and dorsomorphin-treated cells from differentiation day 0 to day 7. Figure S5. NKX2-5 and TBX5 staining of cardiomyocytes in early differentiation. (A) Representative flow cytometry dot plot graphs. (B) Comparison of the percentage of NKX2-5-positive/TBX5-positive cells in total NKX2-5-positive cells (n = 3 in each). Data are shown as means ± standard deviation. (C) Effect of different timings of dorsomorphin or insulin treatment on TBX5 expression in cardiomyocytes. DM: dorsomorphin. Figure S6. Effect of DMH1 on heart field and myocyte subtypes. (A) Immunostaining of TBX5 and NKX2-5 on day 10. (B) Immunostaining of NKX2-5 and cardiac troponin T (cTnT) on day 10. (C) Immunostaining of CXCR4 on day 7. (D) Immunostaining of MLC2v and α-actinin on day 35. The scale bars are 200 µm. (E) Gene expression profiles of purified cardiomyocytes evaluated with quantitative PCR on day 30 (n = 4 in each). Data are shown as means ± standard deviation. (F) Spontaneous contraction frequency of day 33 cardiomyocytes (n = 5 in each). Data are shown as means ± standard deviation. Figure S7. Transcriptomic comparison between control cardiomyocytes and dorsomorphin-treated cell-derived cardiomyocytes. (A) Correlation clustering of induced pluripotent stem cell-derived cardiomyocytes. (B) Correlation heatmap of induced pluripotent stem cell-derived cardiomyocytes. (C) Upregulated genes in the right ventricle compared to in the left ventricle in postnatal day 0 mice. Bold: Upregulated genes in human iPSC-RVCMs compared to iPSC-LVCMs. (D) Downregulated genes in the right ventricle compared to in the left ventricle in postnatal day 0 mice. Bold: Downregulated genes in human iPSC-RVCMs compared to iPSC-LVCMs. Figure S8. Right ventricular-like myocyte induction from commercially available human induced pluripotent stem cell line, 201B7. (A) Immunostaining of CXCR4 and TBX5 on day 7. (B) Immunostaining of NKX2-5 and TBX5 on differentiation day 11. (B) Immunostaining of cardiac troponin T and NKX2-5 on day 11. (C) Immunostaining of MLC2v and α-actinin on day 34. The scale bar is 100 µm. DM: dorsomorphin. cTnT: cardiac troponin T. Figure S9. Effect of genetically inhibition of BMP signaling on cardiomyocyte subtypes. (A) Immunostaining of TBX5, NKX2-5 and cardiac troponin T (cTnT) on day 11. The scale bars are 100 µm. (B) Gene expression profiles on day 11 (n = 3 in each). Data are presented as respective values and their averages.

## Data Availability

The RNA sequencing data have been deposited in the GEO repository with GEO accession number GSE295889. The datasets generated during and/or analyzed during the current study are available from the corresponding author on reasonable request.

## Abbreviations

APA: Action potential amplitude
APD20: Action potential duration at 20% repolarization
APD50: Action potential duration at 50% repolarization
APD70: Action potential duration at 70% repolarization
APD90: Action potential duration at 90% repolarization
bFGF: Basic fibroblast growth factor
GiWi protocol: Cardiac differentiation protocol employing sequential GSK3β inhibition followed by Wnt inhibition
cTnT: Cardiac Troponin T
FHF: First heart field
hESCs: Human embryonic stem cells
hiPSCs: Human induced pluripotent stem cells
hiPSC-CMs: Human induced pluripotent stem cell-derived cardiomyocytes
hPSC-CMs: Human pluripotent stem cell-derived cardiomyocytes
hPSCs: Human pluripotent stem cells
LV: Left ventricle
MDP/RP: Maximum diastolic potential/resting potential
OCR: Oxygen consumption rate
PBMCs: Peripheral blood mononuclear cells
q-PCR: Quantitative polymerase chain reaction
RV: Rght ventricular
RPMI/B27: RPMI 1640 Medium supplemented with B-27 Supplement
RPMI/B27-insulin: RPMI 1640 Medium supplemented with B-27 Supplement minus insulin
SHF: Second heart field
SD: Standard deviation
