# Supplementary material for: Specific induction of right ventricular-like cardiomyocytes from human pluripotent stem cells
Source: Stem Cell Res Ther. 2025 Sep 26;16:519. doi: 10.1186/s13287-025-04656-0 (PMC12465181; doi:10.1186/s13287-025-04656-0)

Supplemental Figure 1

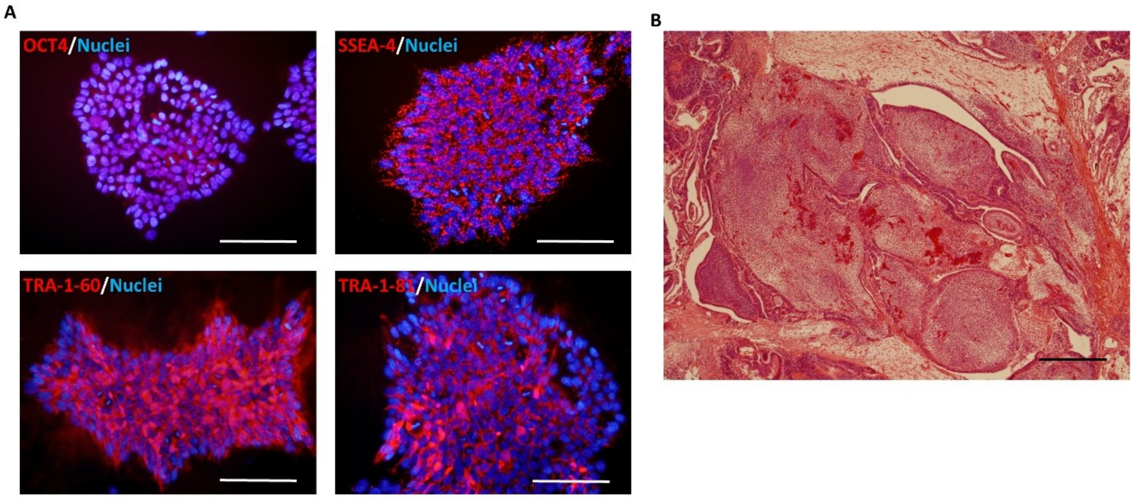

Supplemental Figure 2

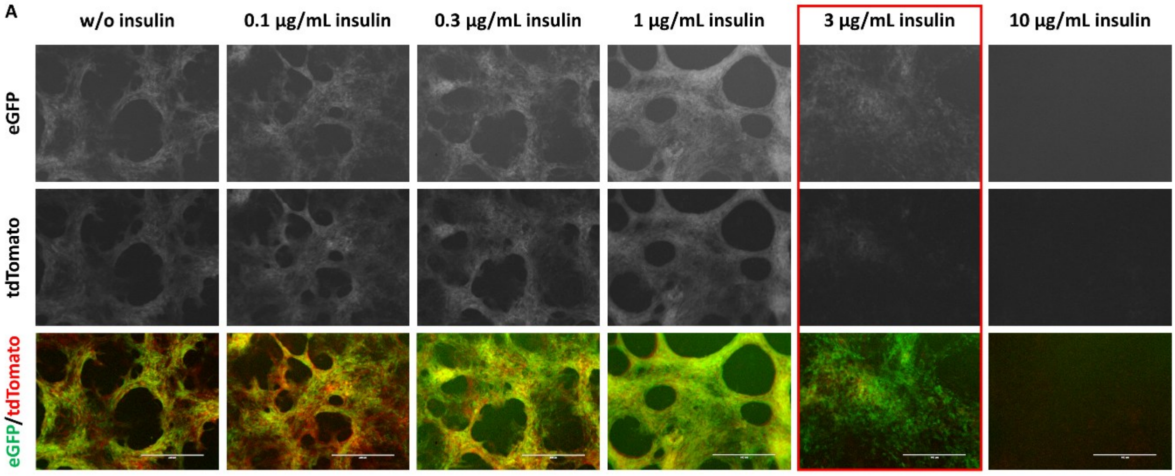

Supplemental Figure 3

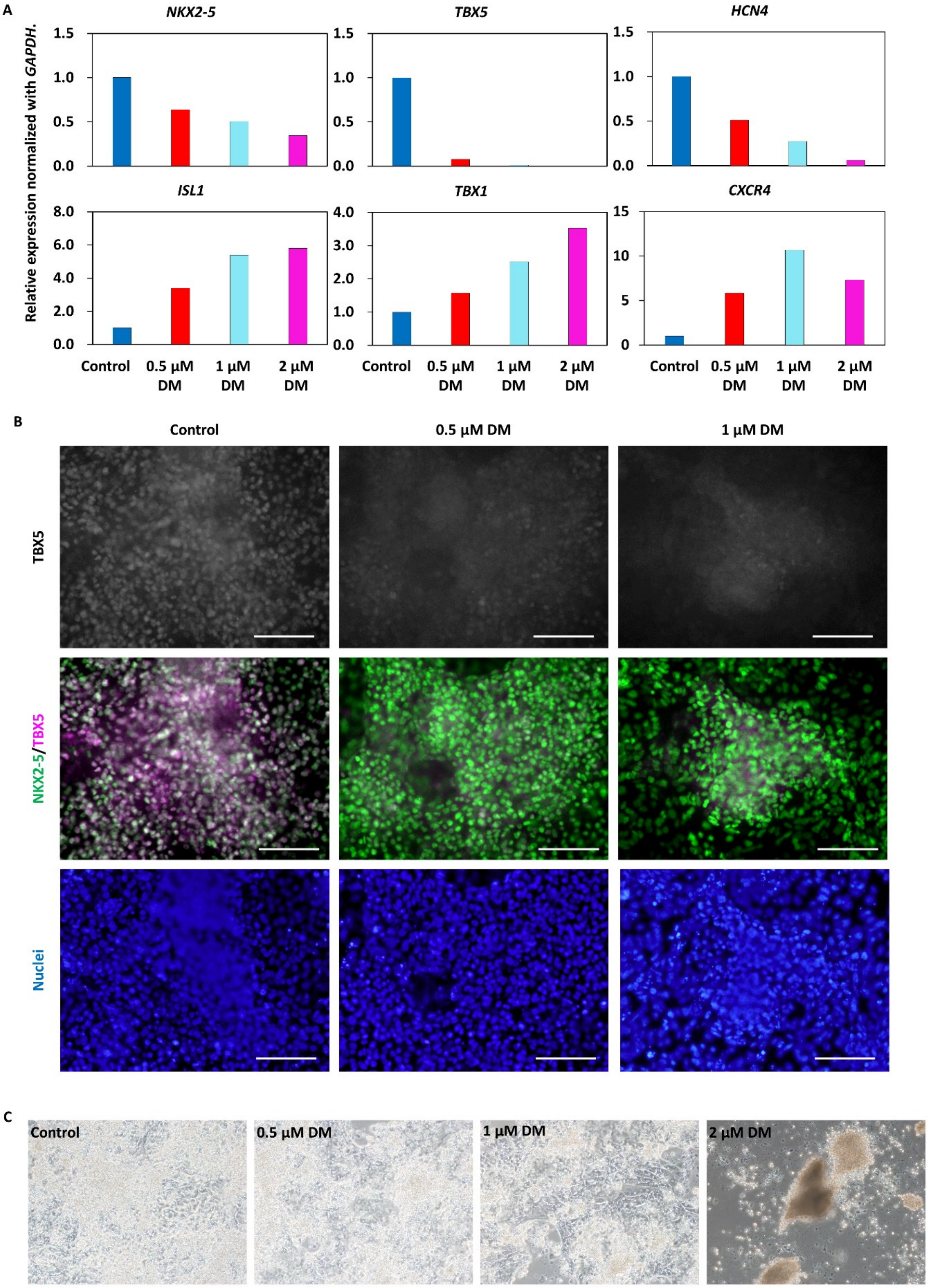

Supplemental Figure 4

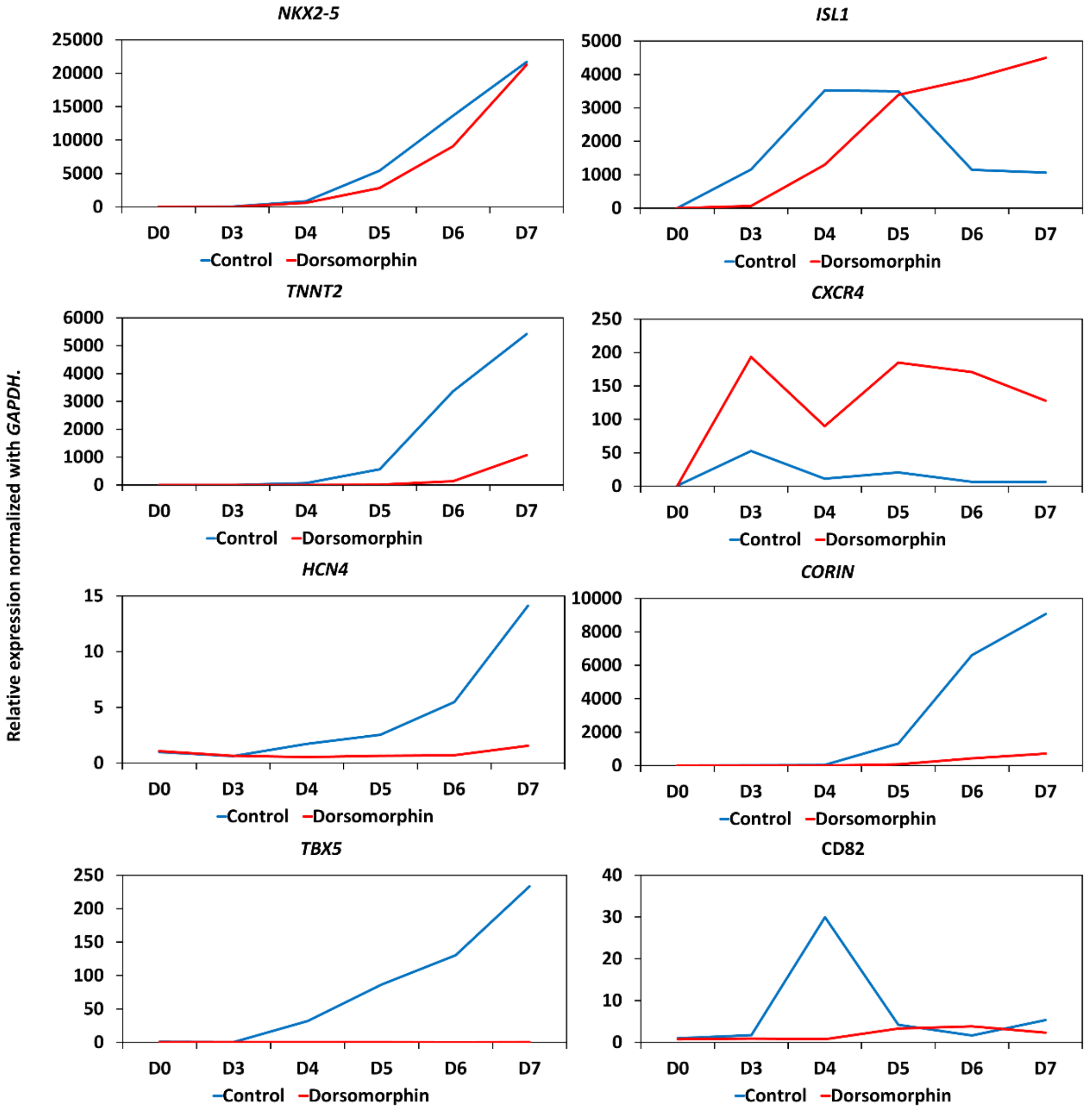

Supplemental Figure 5

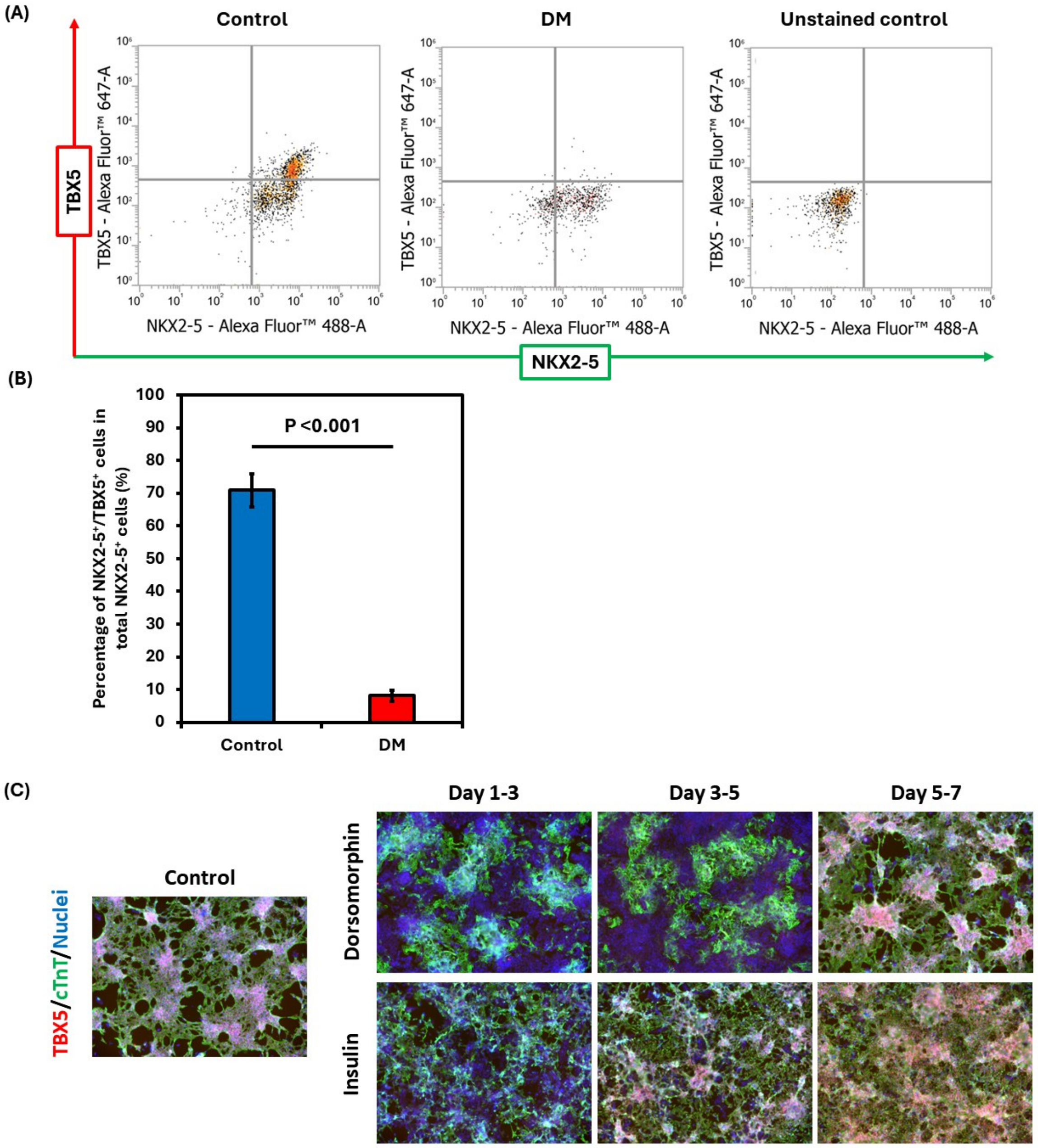

Supplemental Figure 6

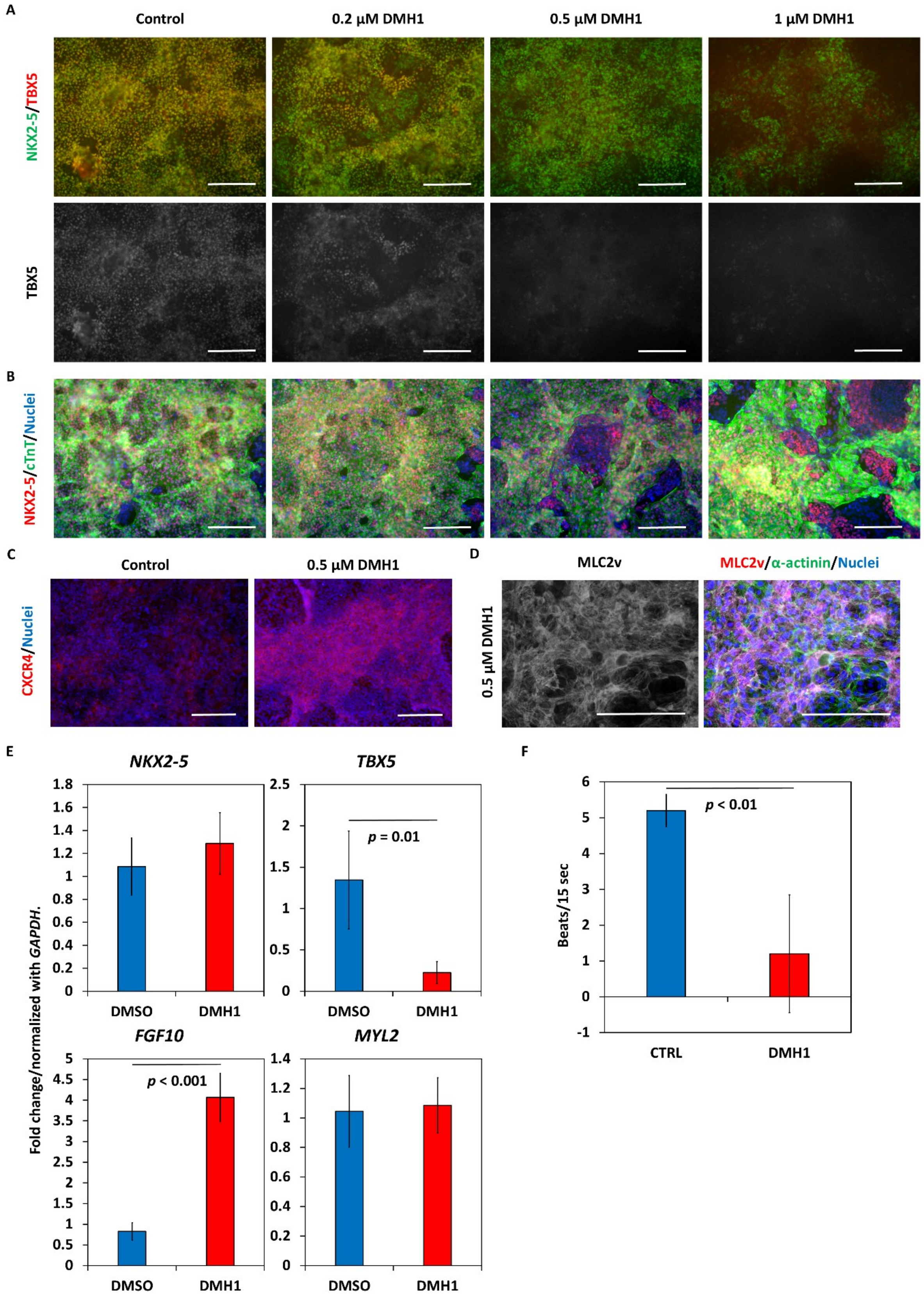

Supplemental Figure 7

A

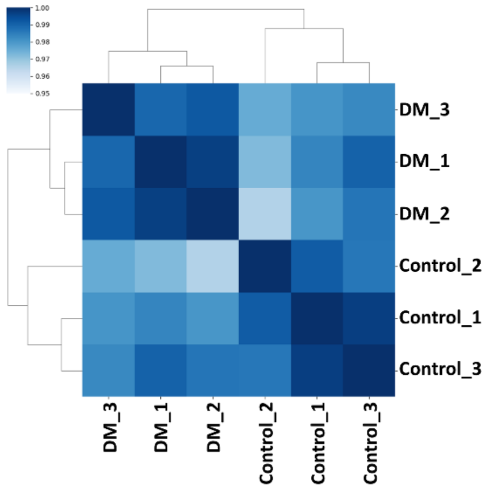

B

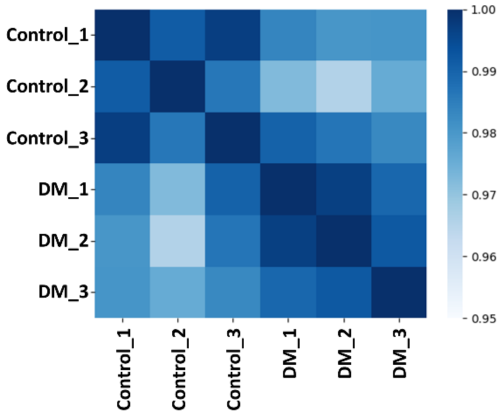

C

Upregulated genes in the right ventricle compared to in the left ventricle in postnatal day 0 mice.

Gene	log_2(Fold Change)	padj
**Ryr3**	**2.084845375**	**2.02472E-07**
**Mybphl**	**1.853537361**	**0.000278595**
**Sln**	**1.699559709**	**6.83926E-13**
Ptgfr	1.444821862	0.013975416
Myh8	1.405347194	0.034801458
**Acta1**	**1.19075729**	**2.69885E-07**
Slc22a1	1.139715767	0.000903365
**Lrtm1**	**1.097893245**	**0.001855866**
Wif1	0.857754107	0.000903365
**Myl7**	**0.846439827**	**0.000188536**
Zfp691	0.834664615	0.002960655
**Cystm1**	**0.809555746**	**0.027240885**
Plvap	0.647285131	0.015749369
Scube2	0.635064133	0.037960114
**Myl1**	**0.588520007**	**0.015749369**
Emcn	0.487783422	0.013975416
Ddc	0.475188139	0.002212693

Bold: Upregulated genes in human iPSC-RVCMs compared to iPSC-LVCMs.

D

Downregulated genes in the right ventricle compared to in the left ventricle in postnatal day 0 mice.

Gene	log_2(Fold Change)	padj
Fig4	-0.480203763	0.001974457
Bub1	-0.497379102	0.048382973
Rnpc3	-0.62197721	0.018546343
Sh3d19	-0.628165269	0.013975416
**Arhgap32**	**-0.632912038**	**0.044385087**
Ppp1r1b	-0.680956476	0.032551057
Nav3	-0.7415426	0.043677046
**Adamts19**	**-0.772354462**	**0.001719004**
**Irx1**	**-0.938327672**	**0.028933781**
**Ntn1**	**-1.027326095**	**4.3802E-07**
**Plekhh1**	**-1.053302515**	**0.000903365**
Nrn1	-1.596539072	0.002960655
Gtsf1	-1.672048974	0.040582157
**Sfrp5**	**-1.972593265**	**0.026567061**
**Hand1**	**-2.267687818**	**1.26529E-08**
Spp1	-4.044028807	0.013975416

Bold: Downregulated genes in human iPSC-RVCMs compared to iPSC-LVCMs.

Supplemental Figure 8

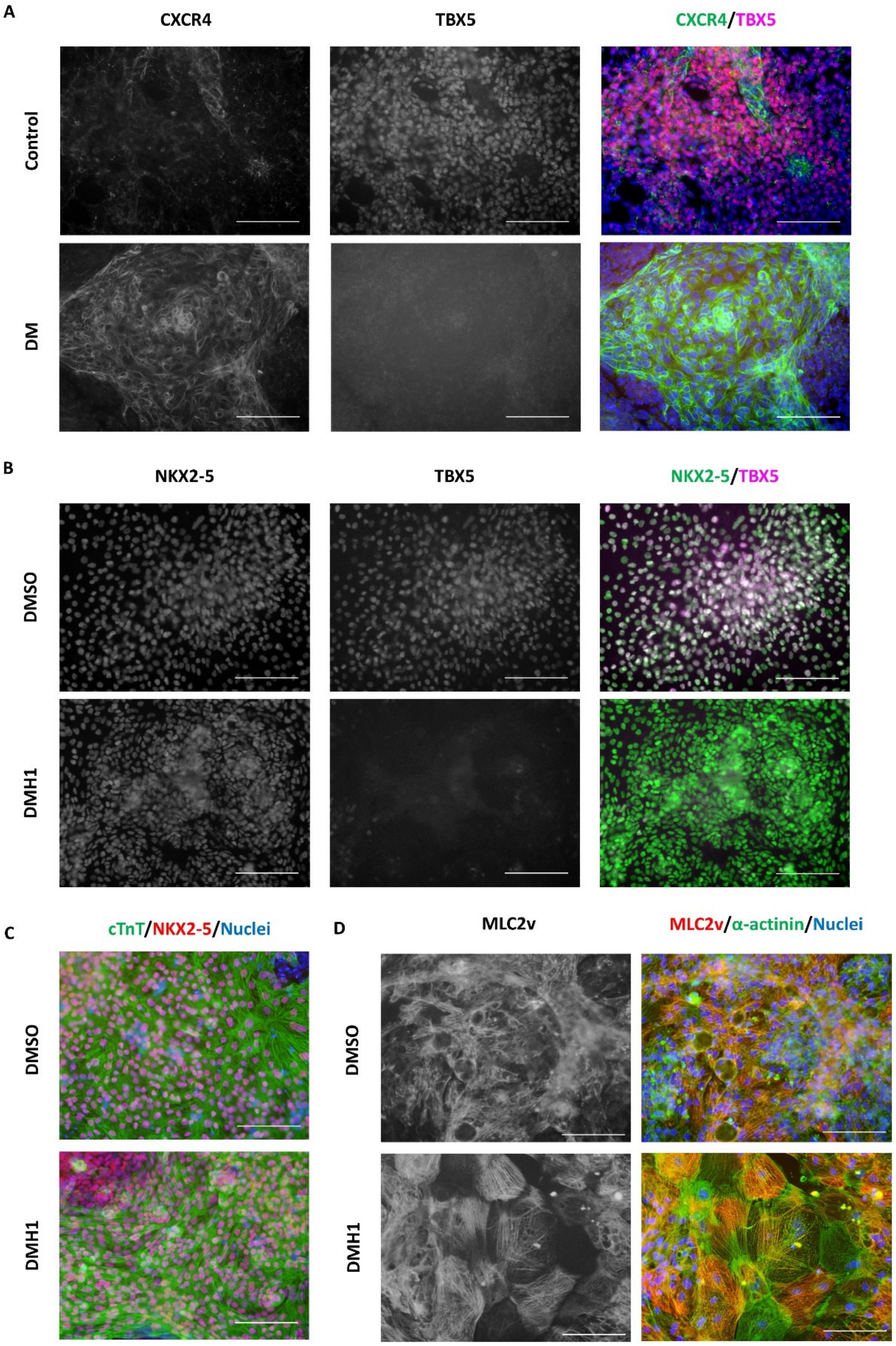

Supplemental Figure 9

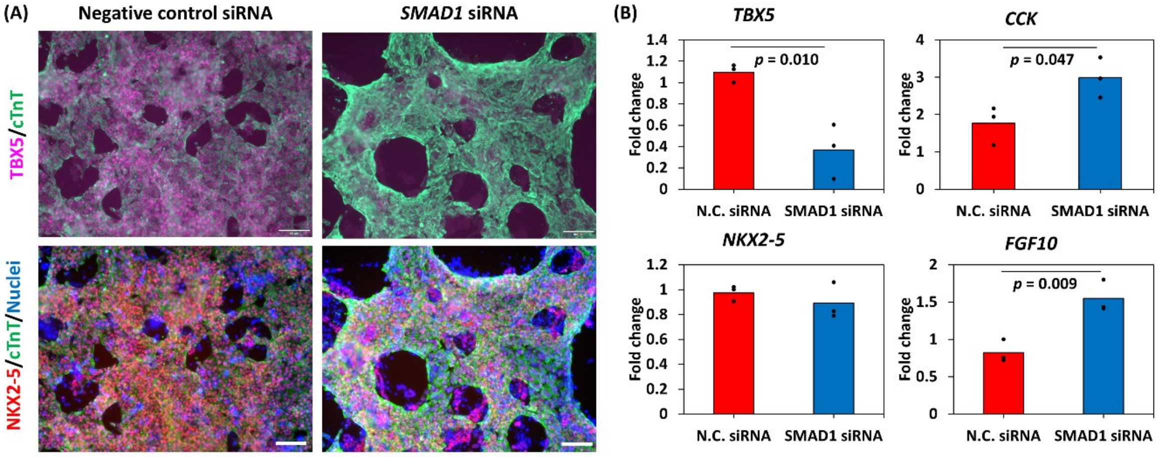

Supplement: Supplementary file 5 — Supplementary Material 5: Figure S1. Characterization of human pluripotent stem cells generated in our laboratory. (A) Immunostaining of undifferentiated cell markers expressed in hiPSCs. Scale bars are 100 µm. (B) Hematoxylin eosin staining of teratoma tissue derived from hiPSCs. The scale bar is 400 µm. Figure S2. (A) Treatment with 3 µg/mL insulin on differentiation day 0 results in low TBX5-tdTomato expression in cardiomyocytes on day 13. The scale bar is 400 µm. Figure S3. Treatment with different concentrations of dorsomorphin on differentiation day 0 induces second heart field-like cells from human induced pluripotent stem cells. (A) Dorsomorphin downregulated the expression of first heart field (FHF) markers and upregulated the expression level of second heart field (SHF) markers on day 7 (n = 1 in each). (B) Dorsomorphin-treated cells expressed NKX2-5, but TBX5 expression was suppressed on differentiation day 10. The scale bar is 100 µm. (C) Phase contrast images of purified cardiomyocytes on differentiation day 20. DM: dorsomorphin. Figure S4. Comparison of gene expression profiles between control and dorsomorphin-treated cells from differentiation day 0 to day 7. Figure S5. NKX2-5 and TBX5 staining of cardiomyocytes in early differentiation. (A) Representative flow cytometry dot plot graphs. (B) Comparison of the percentage of NKX2-5-positive/TBX5-positive cells in total NKX2-5-positive cells (n = 3 in each). Data are shown as means ± standard deviation. (C) Effect of different timings of dorsomorphin or insulin treatment on TBX5 expression in cardiomyocytes. DM: dorsomorphin. Figure S6. Effect of DMH1 on heart field and myocyte subtypes. (A) Immunostaining of TBX5 and NKX2-5 on day 10. (B) Immunostaining of NKX2-5 and cardiac troponin T (cTnT) on day 10. (C) Immunostaining of CXCR4 on day 7. (D) Immunostaining of MLC2v and α-actinin on day 35. The scale bars are 200 µm. (E) Gene expression profiles of purified cardiomyocytes evaluated with quantitative P [file 13287_2025_4656_MOESM5_ESM.pdf]
